# Clinical M2 macrophages-related genes to aid therapy in pancreatic ductal adenocarcinoma

**DOI:** 10.1186/s12935-021-02289-w

**Published:** 2021-10-30

**Authors:** Qianhui Xu, Shaohuai Chen, Yuanbo Hu, Wen Huang

**Affiliations:** 1 The Second Affiliated Hospital, Yuying Children’s Hospital of Wenzhou Medical University, No 109. Xueyuan West Road, Wenzhou, 325000 Zhejiang China; 2 Zhejiang University School of Medicine, Zhejiang Hangzhou, 310009 China

**Keywords:** Pancreatic ductal adenocarcinoma, M2 macrophages, Prognosis prediction, Tumor mutation burden, Tumor immune microenvironment, Clinical therapy

## Abstract

**Background:**

Increasing evidence supports that infiltration M2 Macrophages act as pivotal player in tumor progression of pancreatic ductal adenocarcinoma (PDAC). Nonetheless, comprehensive analysis of M2 Macrophage infiltration and biological roles of hub genes (FAM53B) in clinical outcome and immunotherapy was lack.

**Method:**

The multiomic data of PDAC samples were downloaded from distinct datasets. CIBERSORT algorithm was performed to uncover the landscape of TIME. Weighted gene co-expression network analysis (WGCNA) was performed to identify candidate module and significant genes associated with M2 Macrophages. Kaplan-Meier curve and receiver operating characteristic (ROC) curves were applied for prognosis value validation. Mutation data was analyzed by using “maftools” R package. Gene set variation analysis (GSVA) was employed to assign pathway activity estimates to individual sample. Immunophenoscore (IPS) was implemented to estimate immunotherapeutic significance of risk score. The half-maximal inhibitory concentration (IC50) of chemotherapeutic drugs was predicted by using the pRRophetic algorithm. Finally, quantitative real-time polymerase chain reaction was used to determine FAM53B mRNA expression and TIMER database was utilized to uncover its possible role in immune infiltration of PDAC.

**Results:**

Herein, 17,932 genes in 234 samples (214 tumor and 20 normal) were extracted from three platforms. Taking advantage of WGCNA, significant module (royalblue) and 135 candidate genes were considered as M2 Macrophages-related genes. Subsequently, risk signature including 5 hub genes was developed by multiple analysis, which exhibited excellent prognostic performance. Besides, comprehensive prognostic nomogram was constructed to quantitatively estimate risk. Then, intrinsic link between risk score with tumor mutation burden (TMB) was explored. Additionally, risk score significantly correlated with diversity of tumor immune microenvironment (TIME). PDAC samples within different risk presented diverse signaling pathways activity and experienced significantly distinct sensitivity to administering chemotherapeutic or immunotherapeutic agents. Finally, the biological roles of FAM53B were revealed in PDAC.

**Conclusions:**

Taken together, comprehensive analyses of M2 Macrophages profiling will facilitate prognostic prediction, delineating complexity of TIME, and contribute insight into precision therapy for PDAC.

**Supplementary Information:**

The online version contains supplementary material available at 10.1186/s12935-021-02289-w.

## Introduction

Pancreatic ductal adenocarcinoma (PDAC) as the seventh leading cause of cancer associated death was one of the most common human malignancies globally [1, 2]. There was approximate 459,000 newly diagnosed patients and an almost 432,000 related deaths according to the 2018 global cancer statistics [1]. Given the difficulty of early precision diagnosis and rapid tumor progression, a large number of PDAC cases presented advanced clinical stage or distant metastatic disease at diagnosis [2]. It is of great importance, thus, to develop novel and reliable indicators for prognostic estimation and therapeutic efficacy prediction, further advance tailored therapy.

Cancer immunity harnessed an antitumor immune response to recognize then eliminates the tumor cells through activating the host’s immune system. Currently antitumor immunotherapy attracted people’s interest with the flourish of immune checkpoint inhibitors, but only a minority of cancer patients could benefit from it. Immune checkpoint blockade immunotherapy (i.e., anti-PD-1, etc.,) have made great breakthrough in numerous malignant cancers, however, clinical trials of anti-PD L1 antibodies and CTLA-4 antibodies have been mostly disappointing in PDAC [3, 4]. A primary reason for limited therapeutic efficacy likely lies in extremely immunosuppressive tumor microenvironment [5]. Account for approximately 50% of the tumor cellular population, infiltrating immune cells mostly served as opposing roles in anti-tumor response [6]. There are mounting of myeloid-derived suppressor cells, T cells, tumor correlated fibroblasts and macrophages, in its microenvironment, almost of which significantly inhibited efficient immunotherapy [7]. Among which, M2 Macrophages and M2 Macrophages-associated signaling pathways functioned as pivotal players in suppressing adaptive immunity, facilitating angiogenesis, and accelerating tumor growth [8, 9]. For example, previous study highlighted macrophages were activated into the M2 phenotype to promote the epithelial-mesenchymal transition, invasion, and migration of pancreatic tumor cells [10]. Additionally, High levels of CD163+ M2 macrophages infiltration was reported to be significantly correlated with worse prognosis [11]. The comprehensive analyses focusing on biological roles of M2 Macrophages, however, in prognostic prediction and tumor microenvironment of PDAC remains obscure. Hence, the most reliable and promising strategy for comprehensive evaluation of tumor sensitivity to clinical treatment may be one derived from immune profiles, identifying PDAC cases according to specific risk signatures correlated with M2 Macrophages profiling, generating individualized program to improve efficacy accordingly.

The human gene (Hs Q14153) was named FAM53B (‘family with sequence similarity 53, member B’) by HUGO Gene Nomenclature Committee after its identification by systematic genome data mining. The genome-wide association study of cocaine dependence and related traits identified FAM53B as a risk gene [12]. In addition, FAM53B functioned as crucial regulators in cell proliferation by bounding 14-3-3 chaperones, as well as SKIIP proteins, adaptor proteins connecting DNA-binding proteins to modulators of transcription [13]. However, the possible roles of FAM53B in PDAC were still elusive, it will be of great importance to explore its potential roles in progression of PDAC.

Herein, we amalgamated two PDAC sample datasets, GSE16515 and TCGA-PAAD to investigate the potential role of M2 Macrophages profiling. The M2 Macrophages profiling was obtained by using CIBERSORT algorithm and followed by WGCNA to discovery the most significant module correlated with M2 Macrophages. Next, the candidate genes in the module were further determined using multiple-COX regression model and 5 key genes were finally identified. Then, multi-genes risk model and an integrated prognostic nomogram was developed. The prognostic value was validated in subsequent analysis and external testing group (ICGC-PACA-CA). Moreover, the synergistic effect of risk score with TMB was demonstrated. Additionally, the potential role of risk score in TIME contexture was investigated. Subsequently, the underlying signaling pathways and therapeutic prediction of risk score were investigated. Finally, the biological functions of FAM53B in prognostic prediction, immune infiltration and immunotherapy were further explored to provide robust insights for clinical therapeutic strategy in PDAC. In summary, M2 Macrophages-based risk score was established to serve as robust predictive biomarker and prognostic indicator for clinical outcome prediction, contributing directions to therapeutic management for PDAC.

## Materials and methods

### Collection of muti-omics data

Sequencing profile for PDAC sample together with normal tissues were obtained from TCGA-PAAD project and GSE16515 dataset. The corresponding clinical profiles were also downloaded from the TCGA portal as descripted previously. The R packages limma and sav was employed to perform batch calibration and normalize the expression values among the two platforms. The principal component analysis was employed to validate the normalize result. The RNA sequencing profile of the patients from ICGC-PACA-CA dataset, which contains 195 primary tumor samples, was obtained from ICGC portal (https://dcc.icgc.org/). Next, four categories of somatic mutation data of PDAC patients were obtained from The Cancer Genome Atlas (TCGA) portal. We singled out the mutation files which were obtained through the “SomaticSniper variant aggregation and masking” platform for subsequent analysis. The Human Protein Atlas (http://www.proteinatlas.org) was used to investigate the protein levels of metastatic-related genes.

### Landscape of infiltrating immune cells

With the help of CIBERSORT algorithm (http://cibersort.stanford.edu/), the sequencing data of samples was analyzed and calculated to gain the abundance of 22 tumor-infiltrating immune cells (TICs) subtypes, which represent the cellular constitute of the tumor immune microenvironment [14].

### Weighted gene co-expression network analysis

The sequencing data of the 17,932 genes of the PDAC patients were employed to generate a weight co-expression network using the WGCNA method. The correlations between sample traits and candidate modules are computed to determine the models highly correlated with traits, in which the genes are further analyzed to screen hub genes [15]. In the current study, we employed the immune-infiltrating cells profile, namely CIBERSORT results, as sample phenotypes then select an appropriate soft threshold power (β) value to generate a scaleless network (the index of scale-free topologies = 0.90). Then, similar genes were introduced into the same candidate module employing the “dynamic tree cutting” algorithm when setting the minimum size as 60. Besides, correlation analysis between module characteristic genes and sample traits was implemented by Pearson’s correlation test (*p < 0.05). Finally, we placed the emphasis on the “M2 Macrophages” population and the module most significantly correlated with M2 Macrophages was extracted for subsequent analysis.

### Functional enrichment analysis

Taking advantage of R package “org.Hs.eg.db”, the Entrez ID for each M2 Macrophages related gene was obtained. To elucidate underlying mechanisms of the hub genes related to M2 Macrophages in biological process, we implemented the Kyoto Encyclopedia of Genes and Genomes (KEGG) and Gene ontology (GO) pathways annotation with “clusterProfiler”, “enrichplot” and “ggplot2” packages and visualized the results.

### Construction of M2 macrophages-related prognostic signature

To explore the prognostic role of M2 Macrophages-associated genes, genes from the most significant module were employed to assemble a prognostic risk signature in PDAC. Firstly, candidate genes significantly related with overall survival (p < 0.05) were identified using univariate COX regression analysis. Next, LASSO shrinked all regression coefficients towards 0 and set the coefficients of many irrelevant features exactly to 0 based on the regulation weight λ. The optimal λ was chosen according to the minimum cross‐validation error in 10‐fold cross validation. Then, a multivariate Cox regression model was analyzed to identify hub genes and computed their corresponding coefficients. Finally, prognostic risk model including 11 hub M2 Macrophages‐correlated genes was developed and risk score was calculated as the formula below. Risk score = βgene 1 ×expression level of gene 1 + βgene 2×expression level of gene 2 + · ···· +βgene n × expression level of gene n. Here, β was the regression coefficient in the multivariate Cox regression analysis as described previously [16].

### Validation of the prognostic M2 macrophages-related signature

According to previous risk formula, each PDAC sample obtained corresponding risk score. All samples were stratified into low- and high-risk subgroups when setting the median value of risk scores (1.3001) as the cut-off point. First, K-M survival curve was plotted using R package “survival” to identify prognosis difference. Besides, time-dependent receiver operating characteristic (ROC) curves were analyzed to validate prognostic value. Then, univariate and multivariate Cox regression analysis were performed for validity of risk signature as an independent prognostic indicator. To visualize correlation of risk score with clinicopathological variables, R “pheatmap” package was employed and compared clinical characteristics between low- and high-risk patients.

### Establishment and verification of the nomogram

To identify the optimal prognostic indicator, risk score, age, gender, tumor grade, and clinicopathological stage for 1/2/3-year OS, ROC analysis was performed [17]. To develop a quantitative prognostic pool for PDAC patients, a nomogram plot integrating risk score and other clinicopathological features was constructed to predict 1-, 2‐and 3‐year overall survival rate. Then, we plotted the calibration curve which could present prognostic validity of nomogram.

### Collection and preprocess of epigenetic mutation data

The corresponding somatic alteration information of TCGA-PDAC cohort were obtained from TCGA dataset. TMB was defined as the number of somatic, coding, base replacement, and insert-deletion mutations per megabase of the genome examined using non-synonymous and code-shifting indels under a 5% detection limit. The “maftools” R package [18] was employed to detect the number of somatic non-synonymous point mutations within each sample. The somatic alterations in PDAC driver genes were revealed for samples with low-/high-risk scores.

### Correlation of risk score with TIME characterization

To uncover the correlation between the risk score and tumor infiltrating immune cells, we implemented the seven methods including XCELL, TIMER, QUANTISEQ, MCPcounter, EPIC, CIBERSORT, and CIBERSORT-ABS to evaluate the immune infiltrating situation. Spearman correlation was analyzed to explore the relevance between risk score and the immune infiltration statues. We compared the differences in immune infiltrating cell fraction between low and high-risk subgroups.

The Estimation of Stromal and Immune Cells in Malignant Tumors using Expression Data (ESTIMATE) algorithm [19], as a new algorithm based on the unique properties of the transcriptional profiles, could estimate the tumor cellularity and the tumor purity.

The immune score and stromal score were calculated to quantify the relative enrichment of immune and stromal cells which form the basis for the ESTIMATE score to predict tumor purity.

### Gene set variation analysis

Predominantly, pathway analyses were carried out to evaluate activation of hallmark pathways and metabolic pathways, which were described in the MSigDB databases (https://www.gsea-msigdb.org/gsea/msigdb) [24]. Then, we applied Gene set variation analysis (GSVA) [25] in the GSVA package (version 1.36.3) to assign pathway activity estimates to assess the relative pathway activities in individual samples.

### Prediction of patients’ response to immunotherapy

According to previous research, expression patterns of immune checkpoint blockade-related hub targets might contribute into efficacy of immunotherapy administration [20]. In this study, we fetched 47 immune checkpoint blockade-related genes (i.e., PDCD1, etc.,) and explored their expression levels in risk-low/high samples. To further explore the potential role of risk score in immunotherapeutic prediction, Immunophenoscore (IPS) was used as a novel and robust predictor of response to immunotherapeutic regimens, which quantify the determinants of tumor immunogenicity and characterize the cancer antigenomes and intratumoral immune landscapes [21]. The scoring system was constructed based on a panel of immune-related genes from the four classes: suppressor cells (SC), effector cells (EC), checkpoints or immunomodulators (CP) and MHC-related molecules (MHC). The weighted averaged Z score was computed by averaging the samplewise Z scores of the four classes within the respective category and the sum of the weighted averaged Z score was termed as the IPS.

### Prediction of chemotherapeutic effect

To estimate the sensitivity of chemotherapy, the R package pRRophetic was employed to estimate the half-maximal inhibitory concentration (IC50) of PDAC samples in different ICI score groups. By constructing the ridge regression model based on Genomics of Drug Sensitivity in Cancer (GDSC) (www.cancerrxgene.org/) cell line expression spectrum and TCGA gene expression profiles, the package pRRophetic could estimate IC50 of chemotherapeutic drugs [22].

### Experimental validation

HPNE (human pancreatic cell line) and four human pancreatic cancer cell lines (BxPC-3 cells, PANC-1 cells, and MiaPaCa-2 cells) were purchased from the Cell Bank of the Type Culture Collection of the Chinese Academy of Sciences, Shanghai Institute of Biochemistry and Cell Biology. The cell lines were all cultured in Roswell Park Memorial Institute (RPMI-1640) medium plus 10% fetal bovine serum (FBS; Invitrogen, Carlsbad, CA, USA). All cell lines were grown without antibiotics in a humidified atmosphere of 5% CO_2_ and 99% relative humidity at 37℃. Three different cell lines were subjected to quantitative real-time polymerase chain reaction (qRT-PCR). Quantitative real-time PCR was analyzed as described previously [23]. All samples were analyzed in triplicates. Glyceraldehyde-3-phosphate dehydrogenase (GAPDH) levels were used as the endogenous control and relative expression of FAM53B was calculated using the 2-ΔΔCt method. The sequences of primers used for PCR were as follows: FAM53B, 5′-CCTCAGCATCAGCGACCACAAC-3′ (forward) and 5′-CGGCAACTGGACATCTCATCGG-3′ (reverse); and GAPDH, 5′-CAGGAGGCATTGCTGATGAT-3′ (forward) and 5′-GAAGGCTGGGGCTCATTT-3′ (reverse).

### Statistical analysis

The Wilcoxon test was employed to compare two groups, whereas the Kruskal-Wallis test was carried out to compare more than two groups. Survival curves were analyzed by the Kaplan-Meier log rank test. The chi-square test was performed to correlate the risk score subgroups with somatic mutation frequency, and the Spearman analysis computed the correlation coefficient. CIBERSORT algorithm results with p < 0.05 were adopted for further analysis. Two-tailed p < 0.05 deemed statistical significance. R software (version 4.0.3) was utilized for all statistical analyses.

## Results

### Removing of batch effect

**Fig. 1:**
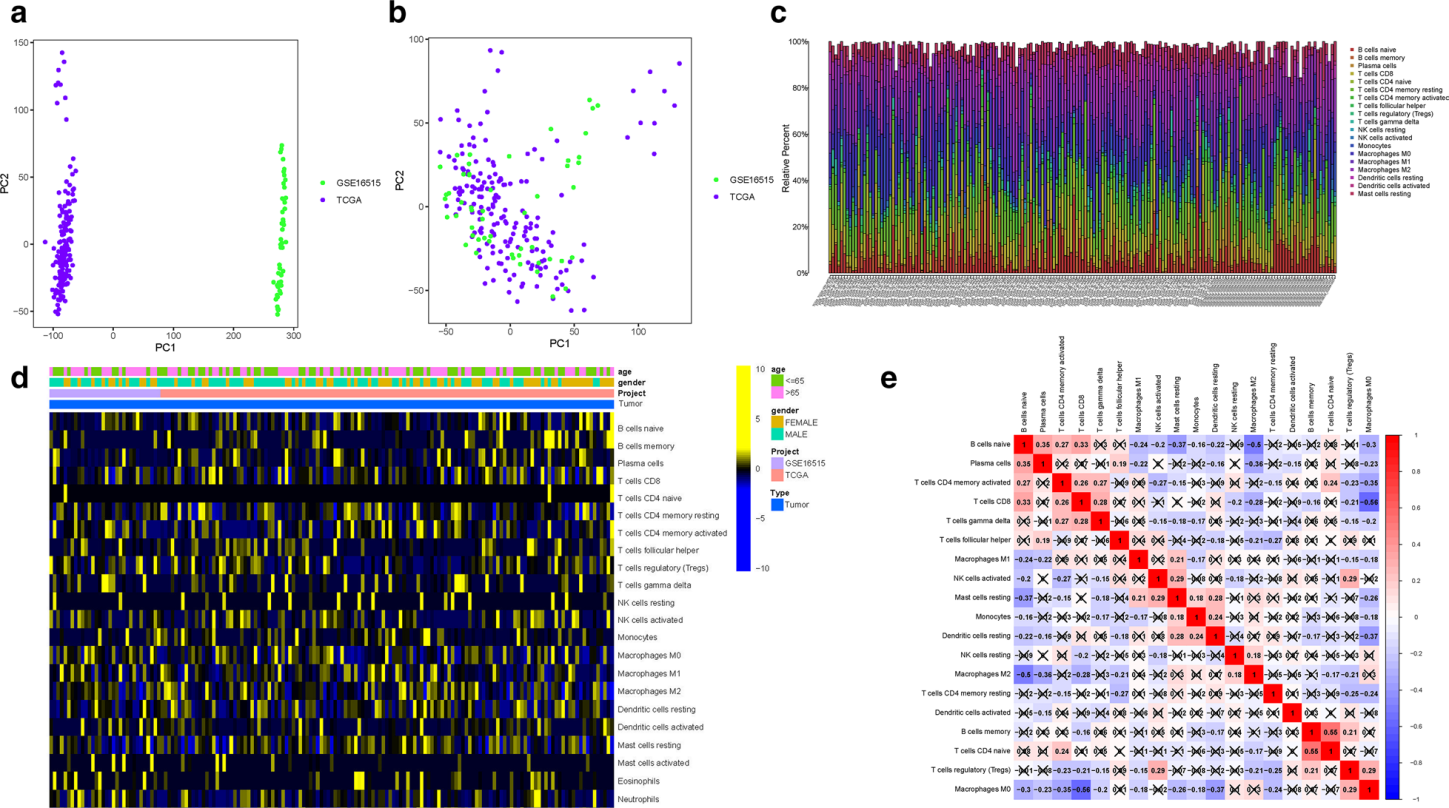
Principal component analysis (PCA) of the gene expression datasets. The points of the scatter plots visualize the samples based on the top two principal components (PC1 and PC2) of gene expression profiles without (**A**) and with (**B**) the removal of batch effect. The colors represent samples from three different datasets, respectively. Landscape of immune cell infiltration in tumor immune environment of PDAC. Subpopulation of 22 immune cell subtypes (**C**) and proportional heatmap of the 22 TICs in each PDAC samples (**D**). **E** Intrinsic correlation of 22 infiltrating immune cells in PDAC. The cross-out cell indicates that the co-expression correlation between two genes is not significant (p > 0.05)

To delete the batch effect in two datasets, the limma and sav algorithm (see Section Method) was employed. A total of 17,932 genes were collectively probed in two different PDAC cohorts (TCGA-PAAD project and GSE16515 microarray). Given the batch effect from different platforms, PDAC samples were gathered by batches based on the top two principal components (PCs) of unnormalized mRNA expression levels (Fig. 1A). After removal of batch effect, the scatter-plot based on principal component analysis (PCA) of normalized sequencing presented that the batch effect was successfully removed by cross-platform normalization (Fig. 1B).

### Landscape of TIME in PDAC

To elucidate the comprehensive landscape of TIME, the CIBERSORT algorithm was employed (Additional file 1: Table S1). Figure 1C presented the abundance of 22 TICs types. The involvement of TIME patterns with clinical phenotypes was explored and depicted in the comprehensive heatmap (Fig. 1D). To further reveal the potential connection between these infiltrating immune cells, the correlation was presented to visualize the comprehensive landscape of TIME (Fig. 1E). Notably, M0 Macrophages was most negatively correlated with CD8+ T cells (r = − 0.56; p < 0.05), whereas naïve CD4+ T cells were most positively correlated with memory B cells (r = 0.55; p < 0.05). When it comes to M2 Macrophages, which was most positively correlated with plasma cells (r = 0.35; p < 0.05), most negative correlation was with M2 Macrophages (r = − 0.5; p < 0.05).

### Establishment of the WGCNA network

**Fig. 2:**
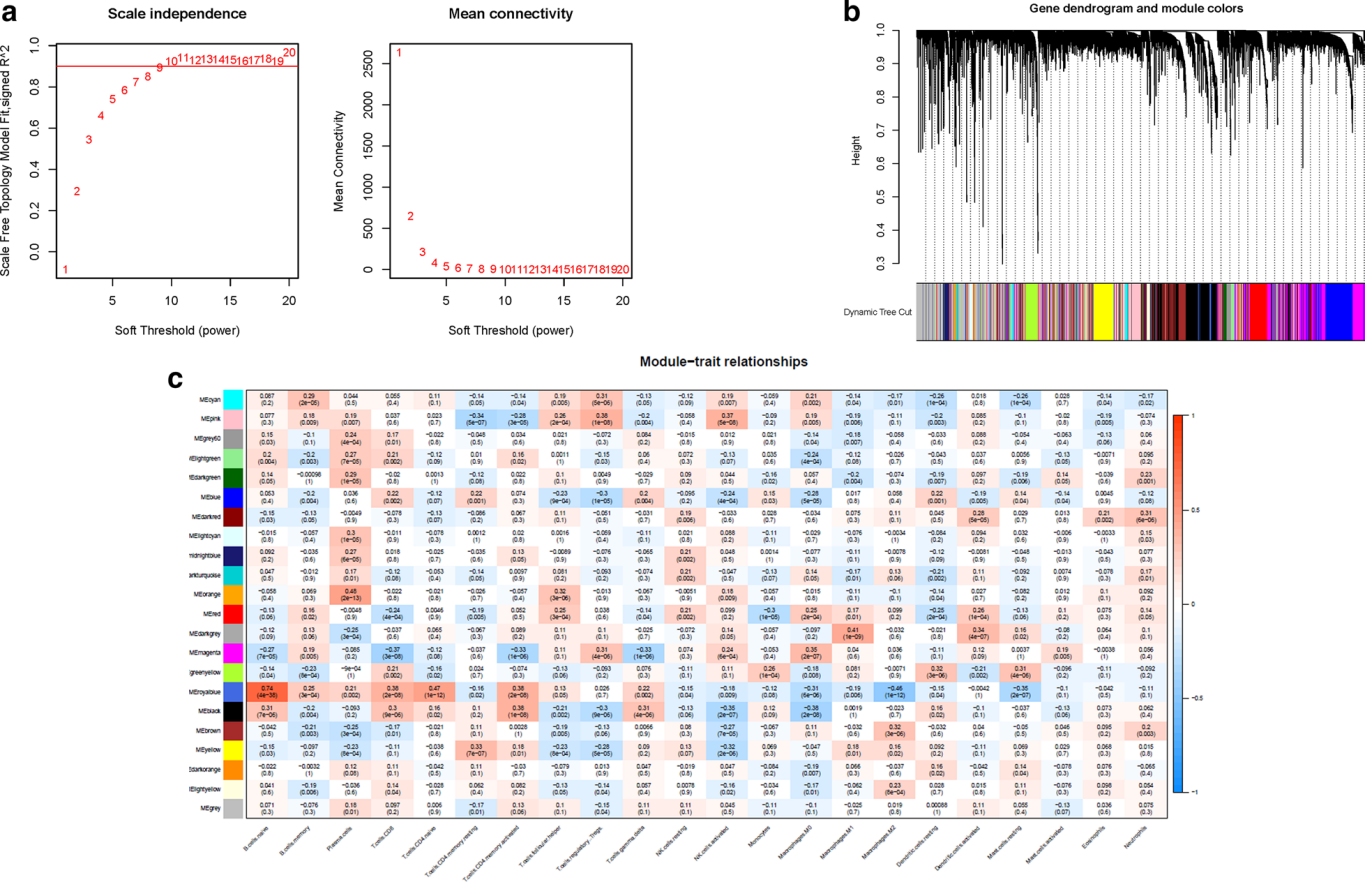
Selection of the appropriate soft threshold (power) and construction of the hierarchical clustering tree. **A** Selection of the soft threshold made the index of scale-free topologies reach 0.90 and analysis of the average connectivity of 1–20 soft threshold power. **B** M2 Macrophages-related genes with similar expression patterns were merged into the same module using a dynamic tree-cutting algorithm, creating a hierarchical clustering tree. **C** Heatmap of the correlations between the modules and immune-infiltrating cells (traits). Within every square, the number on the top refers to the coefficient between the cell infiltrating level and corresponding module, and the bottom is the p value

The sequencing file of 17,932 genes together with the subpopulations of immune infiltration were analyzed to develop the WGCNA co-expression network. In order to construct the scaleless network, the optimal soft threshold power (β) was set as 9 since it was the first power value when the index of scale-free topologies achieve 0.90 (Fig. 2A). Genes with similar expression patterns were introduced into the same module by dynamic tree-cutting algorithm (module size = 60), making a hierarchical clustering tree with different modules. Hierarchical clustering analysis was performed according to weighted correlation, and the clustering results were segmented based on the set criteria to obtain 22 gene modules (Fig. 2B). Each column of Fig. 2C presented the 22 TICs types, and each row presented the candidate module with traits vector genes. It was worth mentioned that the royalblue module was highly correlated with M2 Macrophages (cor = − 0.46, p = 1e−12) among 22 candidate modules. Our primary concern was the M2 Macrophages, and so we fetched the genes (Additional file 1: Table S2) in the royalblue module for further research.

### KEGG and GO enrichment analysis

To explore the biological function of M2 Macrophages-related genes in physiological process, GO and KEGG pathway enrichment were analyzed (Additional file 1: Tables S3 and S4). For KEGG analysis, the top enriched terms were Primary immunodeficiency, Hematopoietic cell lineage and Cytokine−cytokine receptor interaction (Additional file 2: Figure S1A). The result of GO enrichment pathways presented that the M2 Macrophages-related hub genes were mostly enriched in B cell activation, immune response−activating signal transduction and immune response−activating cell surface receptor signaling pathway in biological processes (BP); membrane raft, external side of plasma membrane and membrane microdomain in cellular components (CC); phospholipid binding, RNA polymerase II−specific and DNA−binding transcription activator activity in molecular function (MF; Additional file 2: Figures S1B–D).

### Development of risk signature

**Fig. 3:**
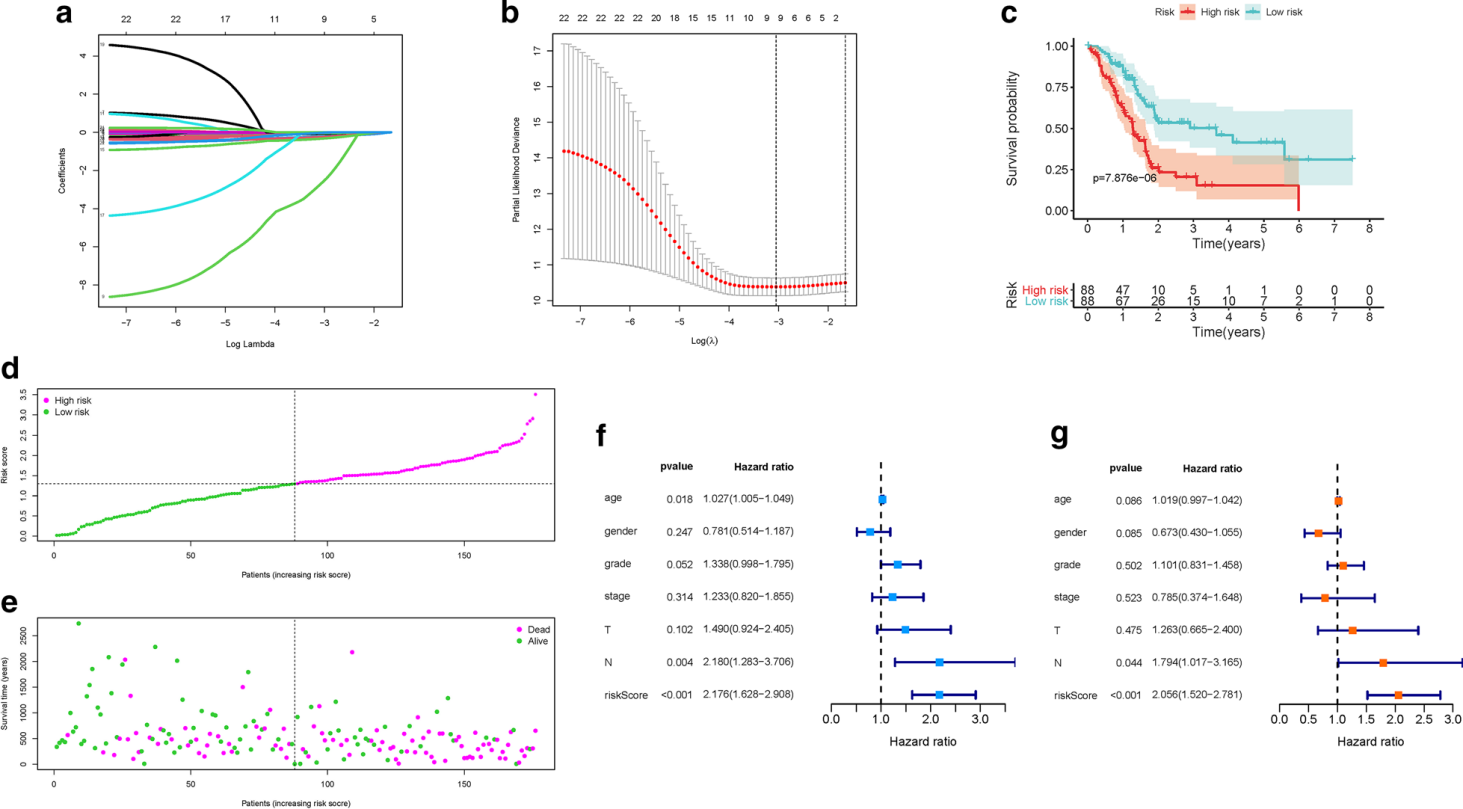
Establishment of the prognostic risk signature. **A** LASSO coefficient profiles of 22 candidate genes. A vertical line is drawn at the value chosen by 10-fold cross‐validation. **B** Ten‐time cross‐validation for tuning parameter selection in the lasso regression. The vertical lines are plotted based on the optimal data according to the minimum criteria and 1-standard error criterion. The left vertical line represents the 9 genes finally identified. **C** Kaplan–Meier curve analysis presenting difference of overall survival between the high-risk and low-risk groups. **D** Distribution of multi-genes model risk score. **E** The survival status and duration of PDAC patients. **F** Univariate Cox regression results of overall survival. **G** Multivariate Cox regression results of overall survival

To further investigate prognostic value of candidate genes, we extracted the expression data and follow-up information from the TCGA-PAAD project. With the help of univariate Cox analysis, 22 M2 Macrophages-related genes were identified with significant prognostic value (p < 0.05, Additional file 1: Table S5). In order to avoid overfitting, the prognostic signature, Lasso regression were conducted on these hub genes and recognized 9 M2 Macrophages-related genes related to prognosis in PDAC (Fig. 3A), and the optimal values of the penalty parameter were determined by 10-round cross-validation (Fig. 3B). Multivariate COX regression analysis was performed, 5 M2 Macrophages-related genes (FAM53B, SPINK2, ABCB4, GH1, INTU) were determined as the hub genes, all of which were considered as beneficial prognostic indicator (all HRs < 1, Table S6). Although, three of these genes (SPINK2, GH1, INTU) have a p-value > 0.05, prognostic accuracy was improved by synergistic effect of them (Additional file 2: Figure S2A–C). Genomics expression value in TCGA database showed that the expression patterns of most genes were abnormally expressed in PDAC tissue compared with normal tissue (Additional file 2: Figure S3A–E). The HPA database was used to explore protein expression levels in PDAC samples. The results showed that relative to normal samples, proteins (ABCB4, FAM53B, and INTU) were significantly dysregulated in tumor tissues (Additional file 2: Figure S4A–J). Furthermore, between low- and high-genes expression subgroups Survival analysis shown that abnormal mRNA expression of most hub genes resulted in significant different overall survival time (most p < 0.05, Additional file 2: Figure S5A–E).

**Fig. 4:**
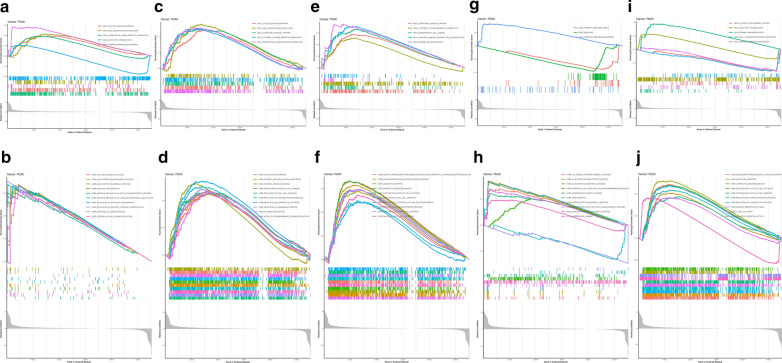
GSEA for samples with high and low expression of 5 hub genes. ** A** The enriched gene sets in KEGG collection by the high ABCB4 expression sample. **B** The enriched gene sets in GO collection by the high ABCB4 expression sample. ** C** The enriched gene sets in KEGG collection by the high FAM53B expression sample. ** D** The enriched gene sets in GO collection by the high FAM53B expression sample. ** E** The enriched gene sets in KEGG collection by the high GH1 expression sample. ** E** The enriched gene sets in GO collection by the high GH1 expression sample. ** F** The enriched gene sets in KEGG collection by the high INTU expression sample. ** G** The enriched gene sets in GO collection by the high INTU expression sample. ** H** The enriched gene sets in KEGG collection by the high SPINK2 expression sample. ** I** The enriched gene sets in GO collection by the high SPINK2 expression sample

According to the median expression of hub genes, all samples were divided into high expression group and low expression group. Then, GSEA was performed to identify the functional enrichment of high and low hub genes expression. KEGG enrichment term exhibited that high expression of ABCB4 was mainly associated with calcium signaling pathway, cell adhesion molecule, and neuroactive ligand receptor interaction (Fig. 4A). Genesets including cortisol metabolism, negative regulation of fatty acid biosynthesis, and negative regulation of vascular endothelial cell proliferation were enriched in patients with high ABCB4 expression (Fig. 4B). The three KEGG signaling pathways most significantly associated with FAM53B high expression were shown in Fig. 4C, where FAM53B high expression was significantly enriched in calcium signaling pathway, cell adhesion molecule, and chemokine signaling pathway. The three GO pathways most significantly associated with FAM53B high expression were shown in Fig. 4D, where FAM53B high expression was positive in humoral immune response, regulation of immune effector process, and regulation of lymphocyte activation. As shown in Fig. 4E, the top 3 KEGG signaling pathways most significantly enriched in GH1 high expression were chemokine signaling pathway, cytokine–cytokine receptor interaction, intestinal immune network for IgA production, respectively. In addition, the GO pathways in leukocyte migration, lymphocyte mediated immunity, regulation of immune effector process were described as the GH1-associated signaling pathways with the greatest enrichment (Fig. 4F). As shown in Fig. 4G, the genes of INTU were mainly enriched in KEGG terms including the primary immunodeficiency and the GO terms including the hormone metabolism, glucocorticoid biosynthesis, glucocorticoid metabolism mainly enriched in the in PDAC (Fig. 4H). The results of GO and KEGG revealed that CDK2 involved in a variety of tumors including Cytosol/DNA/sensing pathway, RIG I Receptor like signaling pathway and immune response regulation signal pathway (Fig. 4I, J).

Subsequently, 5 hub genes were incorporated into a risk signature for PDAC patients. The risk score was computed: risk score = (− 0.0924 ∗ expression value of FAM53B) + (− 0.4320 ∗ expression value of SPINK2) + (− 0.5483 ∗ expression value of ABCB4) + (− 7.7451 ∗ expression value of GH1) + (− 0.5285 ∗ expression value of INTU). Finally, each PDAC sample with corresponding risk score were classified into low-/ high-risk subgroups based on the median cut-off value (1.3001).

### Validation of risk prognostic signature

K–M survival curve demonstrated that high-risk samples presented significant shorter OS time than patients with low-risk (p = 7.876e−06; Fig. 3C). Besides, distributions of dot pot of survival status and risk score suggested that low-risk PDAC patients had longer overall survival time (Fig. 3D, E). Then, univariate Cox analysis pointed out that the hazard ratio (HR) of risk score was 2.176 (95% CI 1.628−2.908; Fig. 3F). And the results of multivariate Cox regression analysis (HR = 2.056, 95% CI 1.520−2.781; Fig. 3G) supported risk score performed as an independent prognostic indicator in PDAC. These results suggested that an excellent capacity of our 5 hub genes signature for clinical outcome prediction.

The signature was employed in the PACA-CA cohort to validate the external prognosis predictive performance. The results presented the distributions of five genes expression patterns, samples survival status, and corresponding risk score in the external validation cohort (Additional file 2: Figure S6A–C). Although there was no statistical significance, survival analysis presented that PDAC patients with high-risk presented poorer prognosis relative to low-risk group patients (Additional file 2: Figure S6D, p = 6.832e−01). The ROC analysis (AUC value = 0.57) also indicated that this risk score model held good prognosis predictive performance in the PACA-CA group (Additional file 2: Figure S6E). These results indicated that the signature had a steady and robust prognostic value. 

### Correlation of risk signature with clinicopathological variables

**Fig. 5:**
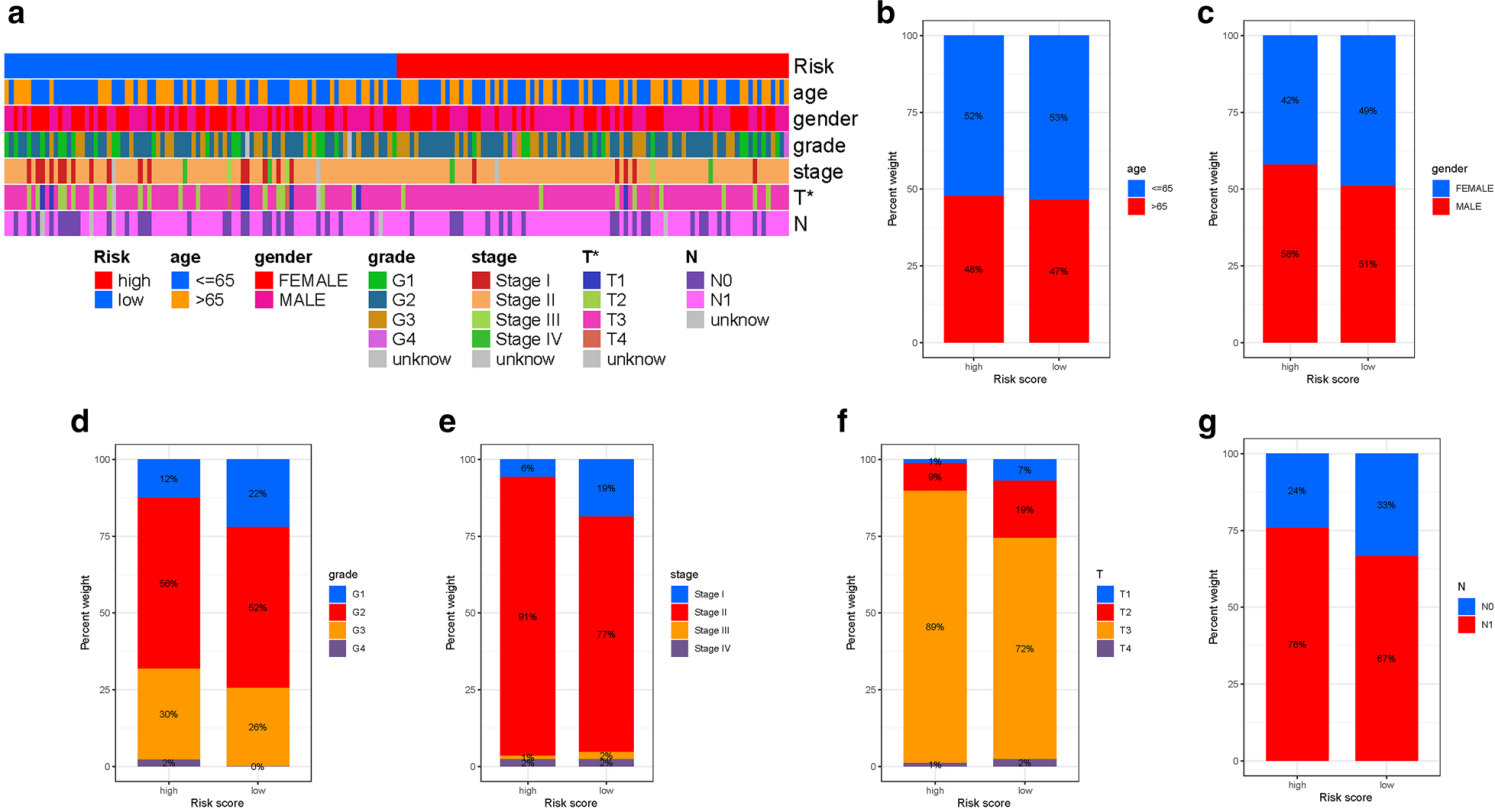
Clinical significance of the prognostic risk signature. **A** Heatmap presents the distribution of clinical feature and corresponding risk score in each sample. Rate of clinical variables subtypes in high or low risk score groups. **B** Age, **C** Gender, **D** WHO grade, **E** clinical stage, **F** T status and **G** N status

Subsequently, the distribution of clinical variables in low/high-risk subgroups was uncovered and visualized (Fig. 5A). Figure 5B–G presented that fraction of clinical subtypes based on age, gender, tumor grade, clinical stage, T status and N category in high-/low-risk subgroup, respectively.

Stratification analysis were employed to validate whether risk score still could identify difference of prognosis when PDAC patients were clustered into clinical variables groups. When patients were divided based on age, we found that our risk score was still predictive of patient outcomes, with higher scores indicating poorer outcomes (Additional file 2: Figure S7A, B). Consistently, risk score presented powerful prognostic predicting ability for patients in male or female gendered (Additional file 2: Figure S7C , D), 1-2 or 3-4 pathological grade patients (Additional file 2: Figure S7E, F), patients in early- and late-stage (Additional file 2: Figure S7G, H), patients T1-2 or T3-4 status (Additional file 2: Figure S7I, J), patients in N0 category (Additional file 2: Figure S7K), and patients in M0 category (Additional file 2: Figure S7L). These findings, combined with results of univariable and multivariable regression analysis, emphasized that our risk score was indeed good prognostic predictive indicator independent from other clinical parameters.

### Construction of prognostic nomogram

**Fig. 6:**
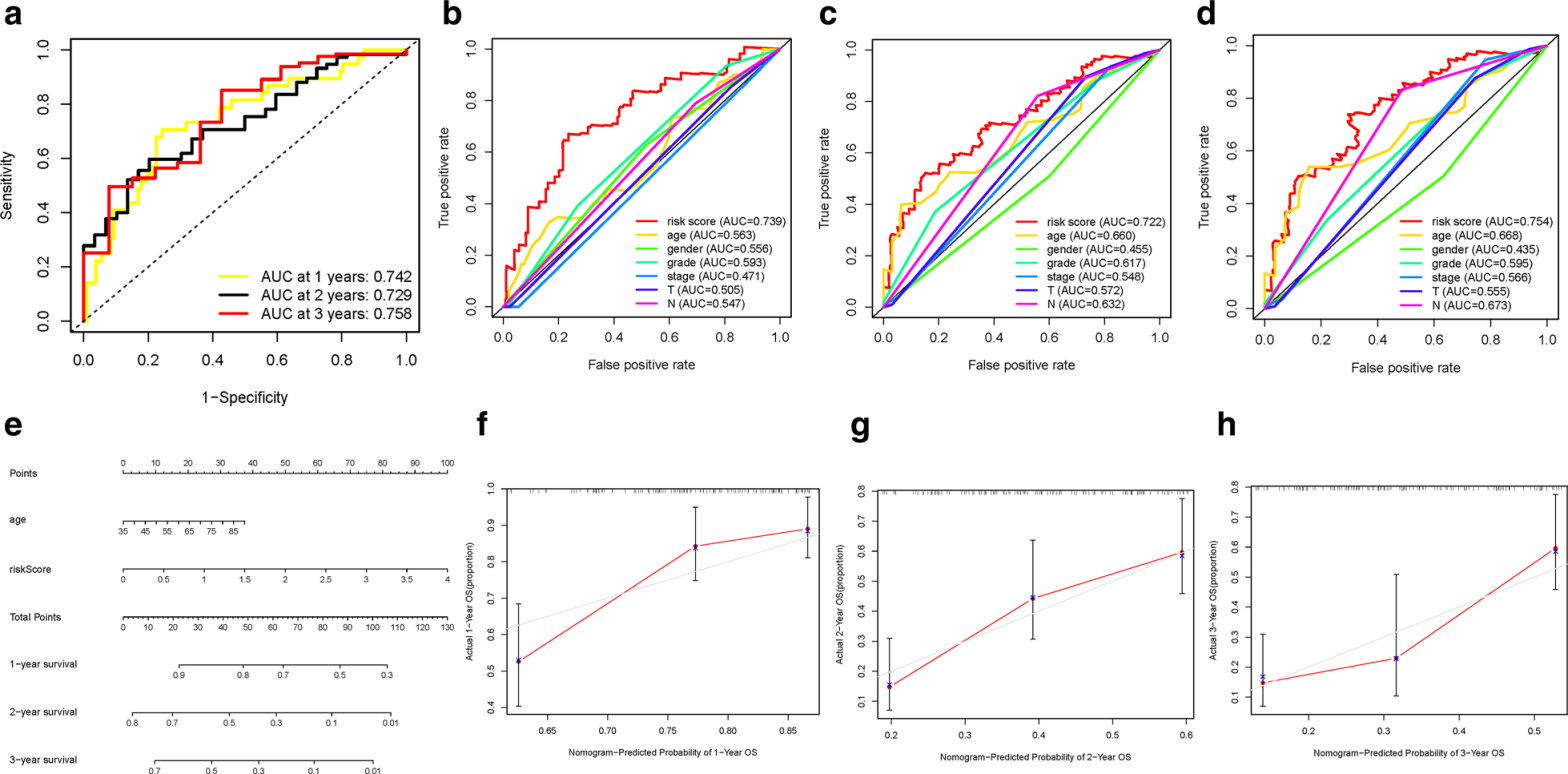
Validation of prognostic efficiency of risk signature. **A** ROC analysis was employed to estimate the prediction value of the prognostic signature. **B–D** Areas under curves (AUCs) of the risk scores for predicting 1-, 2-, and 3-year overall survival time with other clinical characteristics. **E** Nomogram was assembled by stage and risk signature for predicting survival of PDAC patients. **F** One-year nomogram calibration curves. **G** Two‐year nomogram calibration curves. **H** Three‐year nomogram calibration curves

Subsequently, ROC curves were plotted and AUC value for the 1-, 2-, and 3-year OS reached 0.742, 0.729, and 0.758, respectively, suggesting great prognostic validity (Fig. 6A). To further validate risk score was indicator with the best prognostic value among multiple clinicopathological variables, age, gender, clinical staging, tumor grade, T status and N status were assigned as the candidate prognostic factors. These clinical features were incorporated to perform the AUC analysis for 1-, 2-, and 3-year OS and we found that risk score obtained the highest AUC value (Fig. 6B–D). Then, a prognostic nomogram consisting of risk score and clinical stage was developed for quantitative prognosis prediction (Fig. 6E). Gender, stage, tumor grade, T category and N category were excluded out of nomogram given of which AUC values did not reach at 0.6. Finally, calibrate curves suggested excellent prognosis predictive performance of nomogram model (Fig. 6F–H).

### Association of risk signature with TMB

**Fig. 7:**
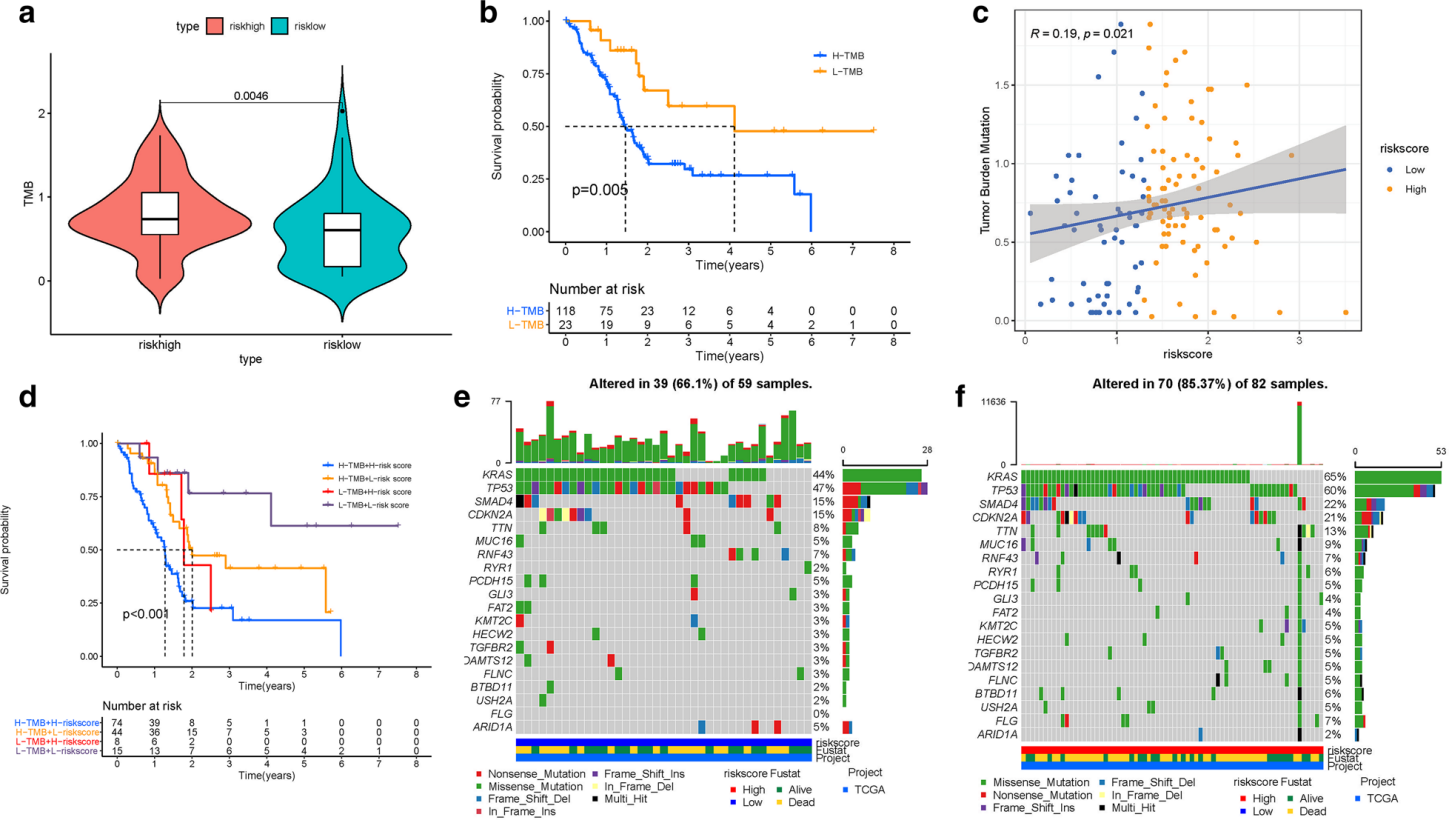
The correlation between the risk Score and TMB. **A** Difference of TMB between patients from the low-/high-risk score subgroups. **B** Kaplan-Meier curves for high and low TMB groups. **C** Scatterplots depicting the positive correlation between risk scores and TMB. **D** Kaplan-Meier curves for patients stratified by both TMB and risk score. The oncoPrint was constructed using high risk score (**E**) and low risk score (**F**)

Recent researches have highlighted that high tumor burden mutation (TMB) was significantly associated with abundance of CD8+ T cells, which could identify cancer cells then leading to anti-tumor immune response [24–26]. For that, we speculated that TMB might act as a nonnegligible prognostic factor of responsiveness to antitumor immunotherapy and aimed to investigate the potential interaction between risk score and TMB to uncover the hereditary variations of risk score subtype. Firstly, the TMB level was detected both in high- and low- risk score subgroups. It was discovered that TMB level was higher in high-risk score subgroup compared with low-risk samples (p = 0.0046, Fig. 7A). Then, the patients were assigned into distinct subtypes on the line of the TMB immune set point, as stated before [27]. Survival curve demonstrated that high TMB value significantly suggested shorter overall survival time (p  = 0.005, Fig. 7B). Subsequent correlation analysis further validated that the TMB was significantly and positively related with the risk score (R = 0.19, p =  0.021; Fig. 7C). To further explore the validity of consistent prognostic significance of risk score and TMB, we validated the cooperative effect of two indicators in prognostic prediction of PDAC. As demonstrated in stratified survival curve, there was no interference of TMB status with risk score prognostic predictive performance. Risk score subgroups exhibited evident prognosis distinctions in both low and high TMB status subtypes (p  < 0.001; Fig. 7D). In summary, these results suggested that risk score might act as independent prognostic predictor and hold the potential to evaluate the clinical outcome of antitumor immunological treatment.

Besides, we explored and visualized the distribution of gene mutation in both the high-and low-risk score subtypes. The comprehensive landscape of somatic variants visualized the mutation patterns and clinical features of the top 20 driver genes with the most frequent alteration (Fig. 7E, F). The significantly mutated gene (SMG) mutational landscapes presented that KRAS (65% vs. 44%) experienced higher somatic mutation rates in high-risk core subtype, while ARID1A (5% vs. 2%) possessed higher somatic mutation rates in the low-risk score subgroup. These findings might contribute novel insight into the intrinsic connection of M2 Macrophages infiltration and somatic variants in immunotherapy of PDAC.

### Risk signature in TIME context of PDAC

**Fig. 8:**
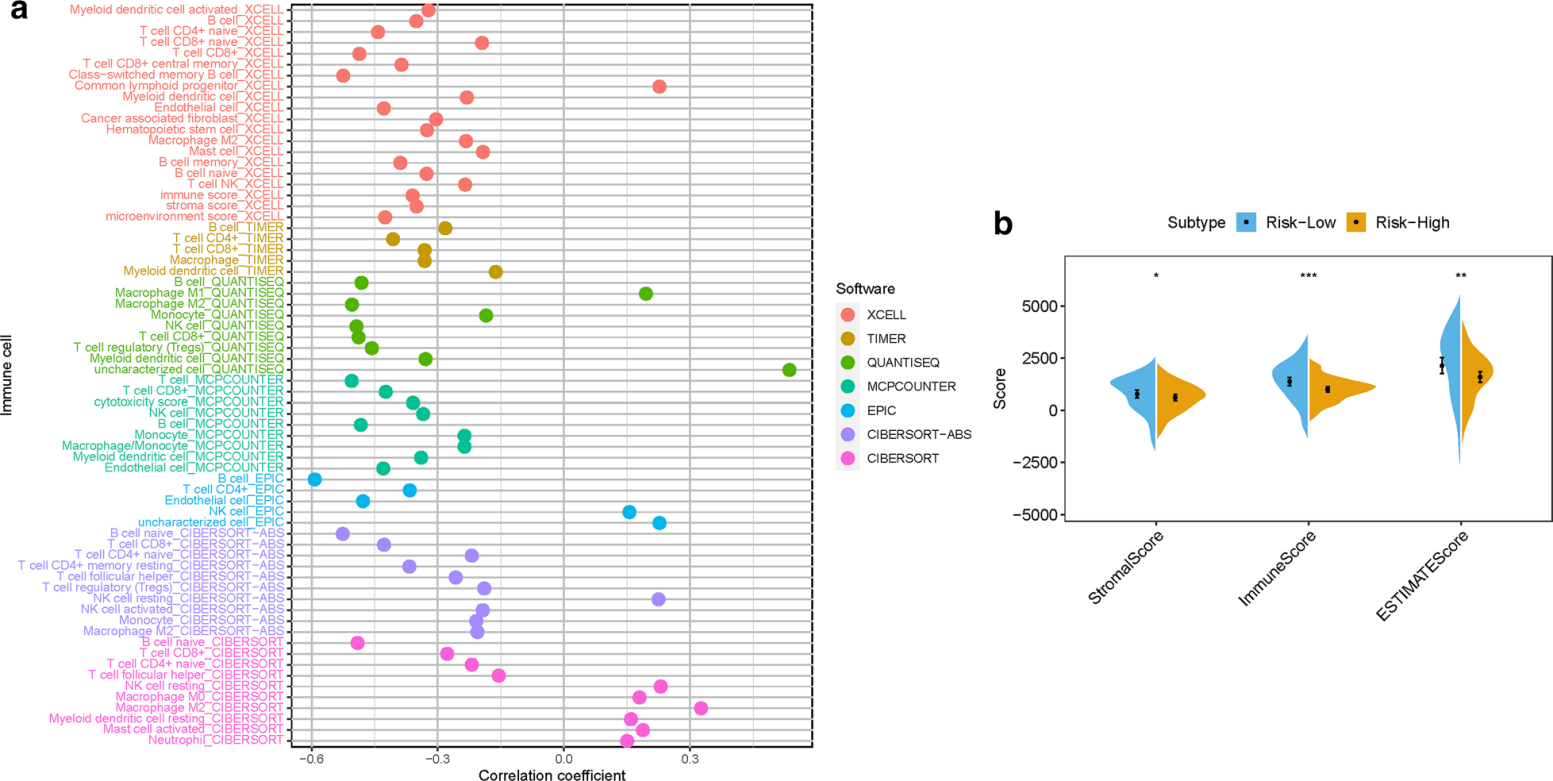
Estimation of abundance of tumor-infiltrating cells. ** A** Patients in the high-risk group were more positively associated with tumor-infiltrating immune cells, as shown by Spearman correlation analysis. Correlation between prognostic risk signature with hub immune checkpoint genes

Since M2 Macrophages-based risk score and infiltration immune cells had intrinsic and intimate connection, we further explored the potential contribution of risk score in complexity and diversity of TIME. The result showed that risk score was negatively and significantly correlated with subpopulations of CD8+ T cells and resting memory CD4+ T cells, while positively correlated with abundance of Monocytes, M0 Macrophages, Endothelial cells and T cell regulatory (Tregs; Additional file 2: Figures S8-S11). Furthermore, Spearman correlation of risk score with immune infiltration was further analyzed (Fig. 8A) and the detailed results were provided in Additional file 1: Table S7. The results of ESTIMATE analysis exhibited that stromal score and immune score experienced significantly higher trend in risk-low group. Likewise, ESTIMATE score was remarkably upregulated in samples with lower risk (Fig. 8B).

### Enrichment of signaling pathways in low/high risk groups

**Fig. 9:**
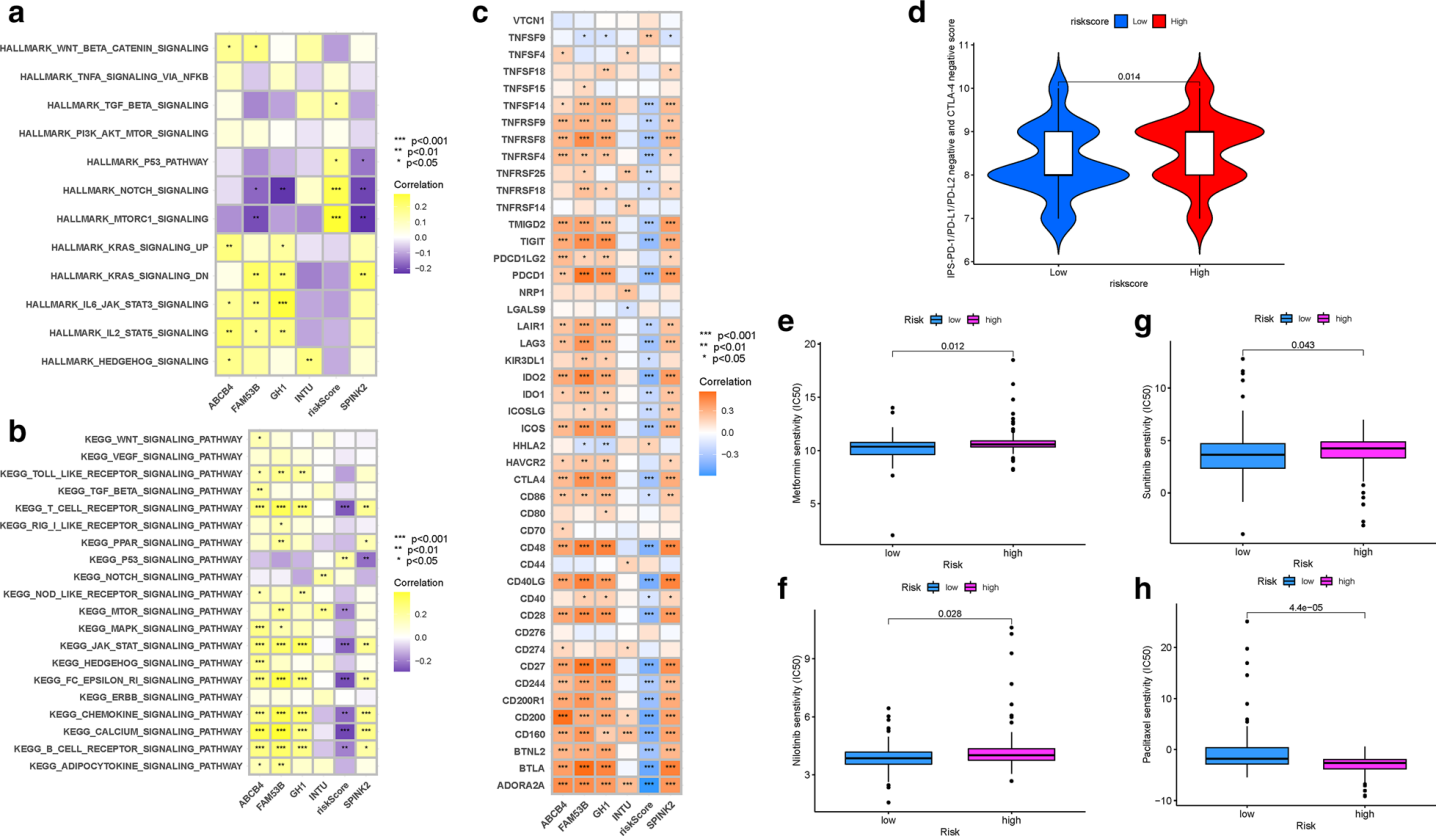
Enrichment pathways of GSVA. **A** Heatmap showing the correlation of representative pathway terms of Hallmark with risk score. **B** Heatmap showing the correlation of representative pathway terms of KEGG with risk score. Prediction of Immunotherapeutic Response. **C** Correlation of expression level of immune checkpoint blockade genes with risk score. **D** IPS score distribution plot. Estimation of Risk Score in Chemotherapeutic Effect. **E** Sensitivity analysis of Metformin in patients at high and low risk score. **F** Sensitivity analysis of Nilotinib in patients at high and low risk score. **G** Sensitivity analysis of Sunitinib in patients at high and low risk score. **H** Sensitivity analysis of Paclitaxel in patients at high and low risk score

To further reveal the biological roles of distinct risk groups in tumorigenicity and progression, gene set variation analysis (GSVA) was performed (Fig. 9A, B). Subjects from lower risk group showed heightened activities of mTOR signaling pathway, JAK/STAT signaling pathway, B cell receptor signaling pathway and T cell receptor signaling pathway. Most genes with high expression levels in high-risk group were enriched in TGF-β signaling pathway, P53 signaling pathway and NOTCH signaling pathway.

### Predicting of patients’ clinical outcome to immunotherapy

Given that the information on immunotherapeutic treatment was not available in TCGA-PAAD dataset, further analysis was explored for response to immunotherapy. Next, it was discovered that most immune checkpoint blockade-related genes (i.e., PDCD1 and CTLA4, etc.) experienced significantly negative correlation with risk score (Fig. 9C). In this risk scoring system, there were no significant differences in the IPS–PD1/PDL1/PDL2 blocker score, IPS–CTLA4 blocker score and IPS–CTLA4 and PD1/PDL1/PDL2 blocker score (Additional file 2: Figure S12A–C). However, high-risk patients possessed higher IPS score (PD-1/PD-L1/PD-L2 negative and CTLA-4 negative; Fig. 9D), suggesting patients with high-risk were more suitable for novel ICB target-based treatment rather than PD1/CTLA4 immunotherapy. Taken together, these results strongly recommend that risk score was correlated with the response to immunotherapies, further predicting prognosis accordingly.

### Prediction of response to chemotherapy

Based on the pRRophetic algorithm, the IC50 of four chemotherapeutic drugs (Metformin, Nilotinib, Paclitaxel, and Sunitinib) were estimated in PDAC patients. Metformin, Nilotinib, and Sunitinib exhibited higher IC50 in patients with high-risk score (all p < 0.05; Fig. 9E, G). In contrary, the IC50 of Paclitaxel was higher in low-risk samples (p = 4.4e−05; Fig. 9H). These results supported the suggestion of patients with different risk score were sensitive to distinct chemotherapeutic drugs.

### The potential role of FAM53B in prognosis, immune infiltration and immunotherapy

**Fig. 10:**
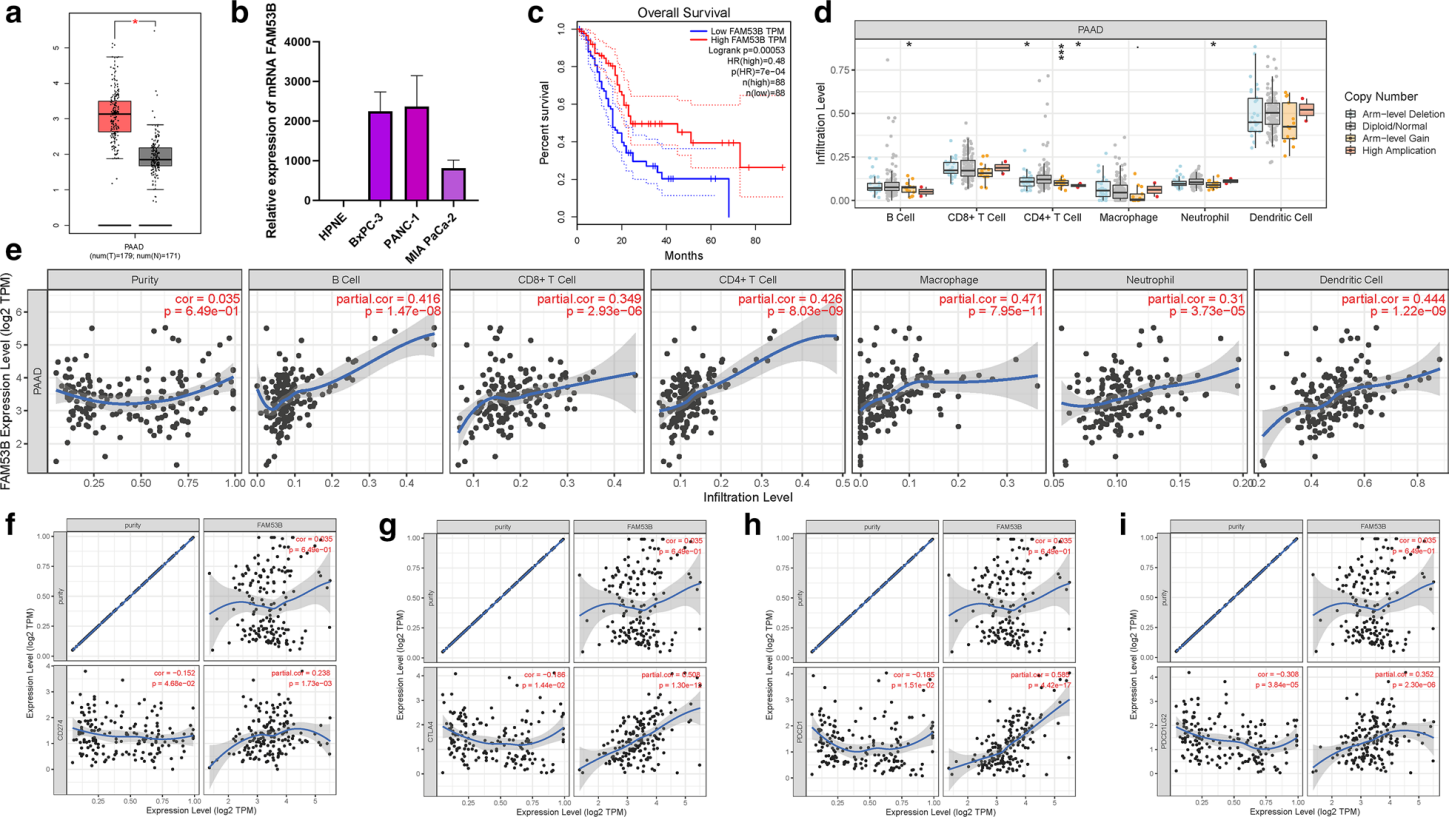
The clinical significance of FAM53B in PDAC. FAM53B are upregulated in PDAC samples based on TCGA dataset (**A**) and cell lines (**B**), and lower FAM53B expression level was significantly correlated with improved prognosis (**C**). **D** Copy number of immune cells in PDAC. **E** Correlation analysis of prognosis-related genes with infiltrating B cells, CD4+T cells, CD8+T cells, Macrophages, Neutrophils and Dendritic cells using TIMER. The association between the expression levels of FAM53B with CD274 (**F**), CTLA4 (**G**), PDCD1 (**H**), and PDCD1LG2 (**I**) using TIMER

FAM53B was hub gene with the most significant dysregulated expression level among these prognostic M2 Macrophages-related genes. For that, the biological function of FAM53B in PDAC was further investigated in subsequent analyses. The expression levels of FAM53B were between tumor samples and normal tissues according to TCGA and GTEx datasets. For tumor tissues and normal specimens, FAM53B expression value exhibited a higher trend in tumor tissues (Fig. 10A). With the help of qRT-PCR, the expression levels of FAM53B in human pancreatic cell line and four distinct pancreatic cancer cell lines were detected. Consistently, normal pancreatic cells presented significantly lower FAM53B values than PDAC cells (Fig. 10B). To estimate the prognostic performance of FAM53B, survival analysis was performed between FAM53B low- and high-expressed samples. It was discovered that lower expression level of FAM53B significantly suggested higher OS rate (P = 0.00053, Fig. 10C). However, there was no significant differences of FAM53B expression between distinct clinical subtypes (i.e., female and male, etc., Additional file 2: Figure S13A–G).

To uncover the potential function of FAM53B in immune infiltration, correlation of FAM53B expression level with infiltrating immune was explored by using TIMER dataset. It was discovered that arm-level gain was predominant type of mutation in immune infiltration (Fig. 10D). Additionally, expression of FAM53B presented significant correlation with B cell (r = 0.416; P = 1.47e−08), CD8+ T cells (r = 0.349; p = 2.93e−06), CD4+ T cells (r = 0.426; p = 8.03e−09), Macrophages (r = 0.471; P = 7.95e−11), Neutrophils (r = 0.31; p = 3.73e−05), and Dendritic cells (r = 0.444; p = 1.22e−09; Fig. 10E).

Subsequently, correlation of FAM53B with immunotherapeutic hub genes adjusted by tumor purity to explore the biological role of FAM53B in immunotherapy. These findings presented that FAM53B experienced significant positive correlation with CD274 (r = 0.238; p = 1.73e−03), CTLA4 (r = 0.508; p = 1.30e−12), PDCD1 (r = 0.585; p = 4.42e−17) and PDCD1LG2 (r = 0.352; p = 2.30e−06; Fig. 10F–I), indicating FAM53B indispensable regulator in immunotherapy of PDAC.

## Discussion

Pancreatic ductal adenocarcinoma (PDAC) is considered as a devastating malignancy and will rank the second leading cause of tumor associated deaths by 2030 [28]. It is common knowledge that such genomic alternation as regulation of non-coding RNA [29], DNA methylation [30] and KRAS mutation [31] served as crucial regulators in PDAC progression. With the rise of immunotherapy, immune checkpoint immunotherapy has significantly revolutionized anticancer therapeutic strategy [32–34]. Existing immunotherapy produce encouraging results in only minority of PDAC cases, however, may be because by immunosuppressive characterization of TIME [35]. M2 Macrophages, functioned as pivotal roles in regulation of antitumor immunity, held promising potential to be next immunotherapeutic target, leading to precision prognostic prediction further advance tailored treatment [36, 37].

More and more emphasis has been placed on infiltrating immune cells in research of human PDAC [38, 39], especially M2 Macrophages. It was well established that M2 Macrophages was considered as the critical players in the immunosuppressive matrix-remodeling, which favor cancer growth [40]. A previous study indicated that high-density M2-Macrophages were significantly correlated with poor prognosis of patients with PDAC [41]. Wang et al. reported that the polarization of M2 macrophages could result in the improved invasiveness of pancreatic tumor cells in vivo and in vitro [10]. These results emphasized that M2 Macrophages may serve as a nonnegligible role in the tumor progression tumor progression, such as immune suppression, cancer initiation and promotion, establishment of premalignant niche and distant metastasis.

In this work, we gathered two distinct PDAC cohort and GSE16515 to explore potential role of M2 Macrophages-related genes in distinct population. A total of 214 tumor samples, and corresponding 17,932 genes were employed in further study. Firstly, CIBERSORT algorithm were performed to obtain the subpopulations of 22 infiltrating immune cells. Next, we determined most significant modules (royalblue) and in which 153 candidate genes positively associated with M2 Macrophages-related genes by using WGCNA method. Additionally, the results of the functional annotation presented that hub genes were mostly enriched in immunological activity and microbial infection, especially B cell activation. Furthermore, it was discovered that abnormal expression value of these genes further remarkedly affected prognosis in PDAC samples, respectively.

To further validate prognostic value of these genes, we fetched the sequencing profile and clinical information from TCGA-PDAC project. Subsequently, we conducted univariate, LASSO and multivariate COX analysis to identify 5 hub genes, then computed risk score and constructed prognostic signature. The excellent prognostic performance of risk model was validated by K-M analysis and ROC curves. We demonstrated that risk signature performed well as an independent prognostic predictor in univariable and multivariable regression analysis. Besides, further validation was analyzed in external dataset (ICGC-PACA-CA cohort). In addition, risk signature remained powerful prognostic ability in clinical variables stratified survival curves. These results suggested that our five-genes risk signature can be applied as an independent prognostic molecular biomarker in predicting clinical outcome for PDAC. Additionally, prognostic risk score-age nomogram was constructed and confirmed to facilitate clinical practice.

Finally, GSEA enrichment was employed to explore the biological roles of FAM53B in tumors and it was discovered that the highly expressed FAM53B was mostly enriched in humoral immune response, regulation of immune effector process, and regulation of lymphocyte activation, and was positive in chemokine signaling pathway. These results indicate that FAM53B is widely involved in the regulation of signaling pathways involved in tumor immunity, providing computational and bioinformatics biology-based insights for further understanding the functions served by FAM53B in anti-tumor strategies.

Currently, several clinical data pointed out a correlation between genetic alternations with responsiveness to immunological treatment [42, 43]. We calculated and determined the TMB, which is a predictive indicator of sensitivity to immunological treatment, increased significantly with risk score elevated. Subsequent stratified survival curve demonstrated that risks score held prognostic predictive capability which was independent of TMB, suggesting that TMB and risk score represent different aspects of immunobiology. Besides, risk score together with mutation data revealed the significant distinction of genes variant frequency between high and low risk score group from the level of transcriptome. In this work, the ARID1A mutation rates were revealed to be markedly augmented in the low-risk score subtype, while the mutation rate of the SMGs of KRAS was increased in the patients with high-risk score. The mutation of KRAS, the major event in PDAC, conferred permanent KRAS activation to activate various transcription factors and signaling pathways [44].

Given risk signature derived from infiltrating immune cells statues, we further investigate the biological function of risk score in TIME characterization and immunotherapy. These findings highlighted that risk score was negatively correlated with subpopulations of activated immune cell (i.e., CD8+ T cells, etc.,), whereas positively correlated with immunosuppressive cells (i.e., Tregs, etc.,), indicating immune activated phenotype of low-risk subgroup with matching OS advantage. Interestingly, higher stromal score was enriched in low-risk group, indicating stromal elements were activated, which could inhibit the antitumor effect of immune cells. By contrary, high-risk samples had relatively low immune scores but more abundance of tumor-promoting immune cells, suggesting immunosuppressive condition of high-risk group. Taken together, these findings highlighted that the stromal activation in low-risk group might suppress an effective antitumor immune response of abundant and activated immune cell infiltration, while the “immune-exhausted phenotype” of high-risk group might lead to immune evasion and immunotherapy resistance.

It was worthy mentioned that GSVA results indicated that mTOR signaling pathway, JAK/STAT signaling pathway were activated in low-risk group, whereas high-risk group were associated with TGF-β signaling pathway, P53 signaling pathway and NOTCH signaling pathway. These results showed that the underlying molecular mechanism diverse well between different risk samples. In addition, risk scoring scheme revealed that sensitivity of chemotherapy drugs was associated with risk score. For that, PDAC patients might be more suitable for distinct combination administration with molecule-targeting and chemotherapeutic agents according to risk stratification.

Moreover, risk score was significantly and negatively associated with ICB-related genes (i.e., PDCD1, etc.,), highlighting samples with low-risk might be more influenced by immune checkpoint blockade. And high-risk group present higher IPS score (PD-1/PD-L1/PD-L2 negative and CTLA-4 negative) and may exhibit a better response to novel target-based (i.e., TIGHT, etc.,) immunotherapy.

Among these M2 Macrophages-related genes in our risk model, the biological functions of FAM53B have not been revealed yet in PDAC. In addition, FAM53B expression was discovered to independently affect OS of patients with PDAC. FAM53B, refers to family with sequence similarity 53, member B, serves as a crucial regulator in the maintenance of a pluripotent state[12]. Recently, accumulating researches focusing on the biological roles of FAM53B in tumors have been published. Such as, a research from Sun et al. indicated that FAM53B accelerated the invasion, migration, and proliferation of ovarian cancer cells, suggesting that FAM53B was an oncogene in ovarian cancer [45]. Qi et al. indicated that FAM53B may act as a critical role to facilitate proliferation and invasion of cancer cells in multiple myeloma (MM) [46]. In this work, prognostic performance and effects on TIME features and immunotherapy of FAM53B were elucidated. It was discovered that FAM53B is significantly overexpressed in PDAC cells and could play as a poor prognostic predictor in PDAC. In addition, FAM53B experienced intimate correlation with immune infiltration (i.e., B cells, etc.,) in PDAC. Moreover, FAM53B expression value exhibited significant positive correlation with immunotherapy hub genes (i.e., CTLA4, and PDCD1, etc.). However, the underlying biomolecular mechanism of FAM53B in PDAC remains obscure, requiring further validation.

Collectively, the landscape of TIME was deciphered by employing distinct datasets and using comprehensive bioinformatic analysis. Besides, the distinction of M2 Macrophages-based risk scoring scheme was demonstrated to contribute to clinical outcome prediction, genes mutation, TIME heterogeneity and therapeutic response. Moreover, the potential role of FAM53B was explored in PDAC. Even though, further experimental and clinical validation were required for these findings at different centers and larger cohort.

## Supplementary Information

**Additional file 1:** **Table S1**: The Results of CIBERSORT algorithm. **Table S2**: The genes and corresponding modules after WGCNA. **Table S3**: The results of GO analysis of naïve B cells-related genes. **Table S4**: The results of KEGG analysis of naïve B cells-related genes. **Table S5**: The results of univariate regression analysis. **Table S6**: The results of multivariate regression analysis. **Table S7**: The results of correlation of risk score with immune infiltrating cell.

**Additional file 2:** **Figure S1**: Pathway enrichment analyses of M2 Macrophages-related genes. Gene Ontology (GO) enrichment analysis of M2 Macrophages-related genes: biological processes (BP) (A), cellular components (B) and molecular function (C). (D) KEGG enrichment analysis of M2 Macrophages-related genes. **Figure S2**: ROC analysis of prognostic signature and five hub genes. (A) Areas under curves (AUCs) of the risk scores for predicting 1-year overall survival time with five hub genes. (B) Areas under curves (AUCs) of the risk scores for predicting 2-year overall survival time with five hub genes. (C) Areas under curves (AUCs) of the risk scores for predicting 3-year overall survival time with five hub genes. **Figure S3**: The mRNA expression level of hub genes in TCGA cohort. (A) ABCB4, (B) FAM53B, (C) GH1, (D) INTU, (E) SPINK2. **Figure S4**: Differentially expressed proteins of ABCB4 in normal (A) and pancreatic cancer tissues (B) in the Human Protein Atlas database. Differentially expressed proteins of FAM53B in normal (C) and pancreatic cancer tissues (D) in the Human Protein Atlas database. Differentially expressed proteins of GH1 in normal (E) and pancreatic cancer tissues (F) in the Human Protein Atlas database. Differentially expressed proteins of INTU in normal (G) and pancreatic cancer tissues (H) in the Human Protein Atlas database. Differentially expressed proteins of SPINK2 in normal (I) and pancreatic cancer tissues (J) in the Human Protein Atlas database. **Figure S5**: Survival analysis between high- and low- expression groups of hub genes. (A) ABCB4, (B) FAM53B, (C) GH1, (D) INTU, (E) SPINK2. **Figure S6**: Confirmation of prognostic risk scores in the ICGC cohort. (A) Heatmap of the 5 hub genes expression in PDAC. The color from red to green shows a trend from high expression to low expression. (B) Distribution of model risk score. (C) The survival status and duration of PDAC patients. (D) Kaplan–Meier curve analysis presenting difference of overall survival between the high-risk and low-risk subgroups. (E) ROC analysis of the risk scores for prognosis prediction. **Figure S7**: Kaplan–Meier survival analysis for multiple PDAC subgroups according to the risk signature stratified by clinical variables. (A-B) Age. (C-D) Gender. (E-F) Tumor grade. (G-H) Stage. (I-J) T status. (K) N status. (L) M status. **Figure S8-S11**: The representative results of the evaluation of tumor infiltrating immune cells with risk signature. **Figure S12**: Prediction of Immunotherapeutic Response. (A) IPS–CTLA4 blocker score distribution plot. (B) IPS–PD1/PDL1/PDL2 blocker score distribution plot. (C) IPS–CTLA4 and PD1/PDL1/PDL2 blocker score distribution plot. **Figure S13**: The clinical significance of FAM53B in PDAC. Distribution of risk score in distinct clinical variables subtypes. (A) Age, (B) Gender, (C) WHO grade, (D) clinical stage, (E) T status, (F) N status and (G) M status.

## Data Availability

The datasets generated for this study can be found in the TCGA database (https://portal.gdc.cancer.gov) and GEO database (https://www.ncbi.nlm.nih.gov/geo/).

## Abbreviations

AUC: Area under the curve
BP: Biological processes
CC: Cellular components
CD274: Also known as PD-L1
CI: Confidence Interval
CTLA-4: Cytotoxic T‐lymphocyte antigen 4
CTLs: Cytotoxic T lymphocytes
DCs: Dendritic cells
DEL: Deletion
FDR: False discovery rate
GDSC: Genomics of Drug Sensitivity in Cancer
GO: Gene ontology
GSEA: Gene set enrichment analysis
GSVA: Gene set variation analysis
HR: Hazard ratio
ICB: Immune checkpoint blockade
IDO1: Indoleamine 2,3-dioxygenase 1
IPS: Immunophenoscore
KEGG: Kyoto Encyclopedia of Genes and Genomes
K-M: Kaplan-Meier
K-W: Kruskal-Wallis
LASSO: Least absolute shrinkage and selection operator
MF: Molecular function
OS: Overall survival
PDAC: Pancreatic ductal adenocarcinoma
PDCD1: Also known as PD-1
PDCD1LG2: Also known as PD-L2
ROC: Receiver operating characteristic
TCGA: The Cancer Genome Atlas
TICs: Tumor-infiltrating immune cells
TILs: Tumor Infiltrating Lymphocytes
TIM-3: T‐cell immunoglobulin domain and mucin domain‐containing molecule‐3
TIME: Tumor immune microenvironment
TMB: Tumor mutation burden
TNM: Tumor Node Metastasis
Tregs: Regulatory T cells
WGCNA: Weighted gene co-expression network analysis
