# Supplementary material for: Clinical M2 macrophages-related genes to aid therapy in pancreatic ductal adenocarcinoma
Source: Cancer Cell Int. 2021 Oct 30;21:582. doi: 10.1186/s12935-021-02289-w (PMC8557582; doi:10.1186/s12935-021-02289-w)
Supplement: Supplementary file 2 — Additional file 2: Figure S1: Pathway enrichment analyses of M2 Macrophages-related genes. Gene Ontology (GO) enrichment analysis of M2 Macrophages-related genes: biological processes (BP) (A), cellular components (B) and molecular function (C). (D) KEGG enrichment analysis of M2 Macrophages-related genes. Figure S2: ROC analysis of prognostic signature and five hub genes. (A) Areas under curves (AUCs) of the risk scores for predicting 1-year overall survival time with five hub genes. (B) Areas under curves (AUCs) of the risk scores for predicting 2-year overall survival time with five hub genes. (C) Areas under curves (AUCs) of the risk scores for predicting 3-year overall survival time with five hub genes. Figure S3: The mRNA expression level of hub genes in TCGA cohort. (A) ABCB4, (B) FAM53B, (C) GH1, (D) INTU, (E) SPINK2. Figure S4: Differentially expressed proteins of ABCB4 in normal (A) and pancreatic cancer tissues (B) in the Human Protein Atlas database. Differentially expressed proteins of FAM53B in normal (C) and pancreatic cancer tissues (D) in the Human Protein Atlas database. Differentially expressed proteins of GH1 in normal (E) and pancreatic cancer tissues (F) in the Human Protein Atlas database. Differentially expressed proteins of INTU in normal (G) and pancreatic cancer tissues (H) in the Human Protein Atlas database. Differentially expressed proteins of SPINK2 in normal (I) and pancreatic cancer tissues (J) in the Human Protein Atlas database. Figure S5: Survival analysis between high- and low- expression groups of hub genes. (A) ABCB4, (B) FAM53B, (C) GH1, (D) INTU, (E) SPINK2. Figure S6: Confirmation of prognostic risk scores in the ICGC cohort. (A) Heatmap of the 5 hub genes expression in PDAC. The color from red to green shows a trend from high expression to low expression. (B) Distribution of model risk score. (C) The survival status and duration of PDAC patients. (D) Kaplan–Meier curve analysis presenting difference of overall survival betw [file 12935_2021_2289_MOESM2_ESM.pdf]

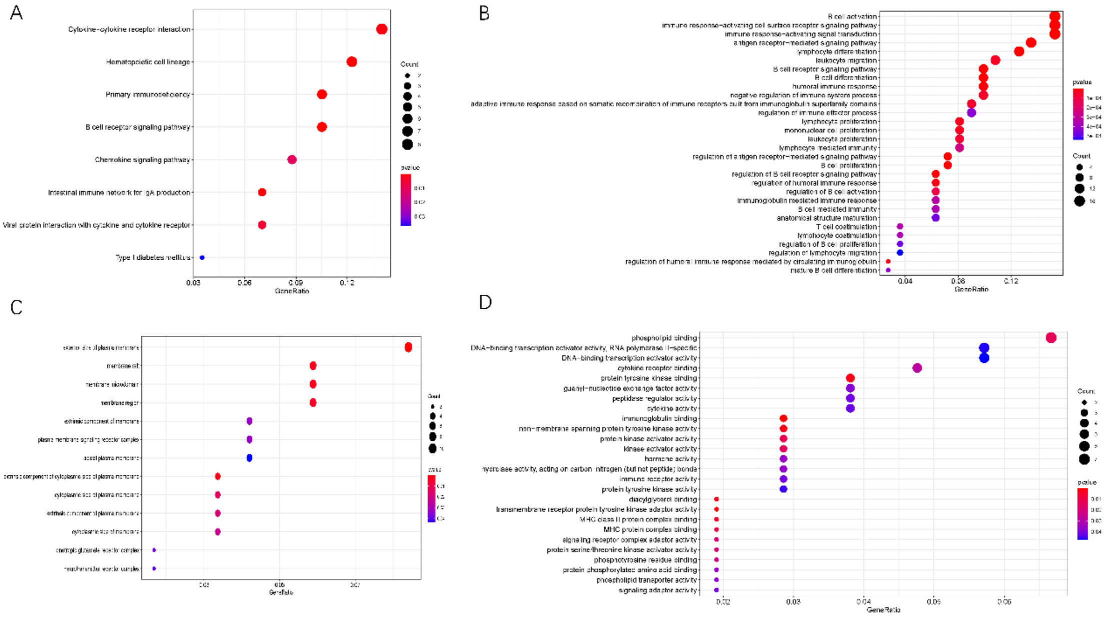

Figure S1: Pathway enrichment analyses of naïve B cells-related genes. Gene Ontology (GO) enrichment analysis of naïve B cells-related genes: biological processes (BP) (A), cellular components (B) and molecular function (C). (D) KEGG enrichment analysis of naïve B cells-related genes.

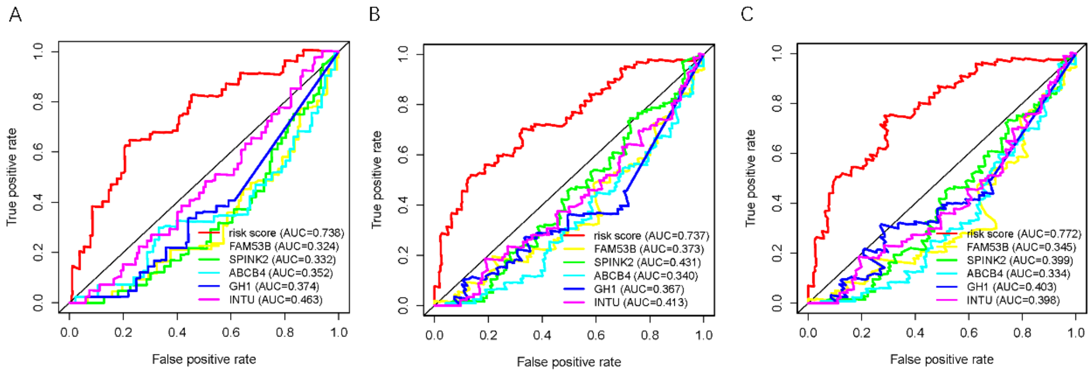

Figure S2: ROC analysis of prognostic signature and five hub genes. (A) Areas under curves (AUCs) of the risk scores for predicting 1-year overall survival time with five hub genes. (B) Areas under curves (AUCs) of the risk scores for predicting 2-year overall survival time with five hub genes. (C) Areas under curves (AUCs) of the risk scores for predicting 3-year overall survival time with five hub genes.

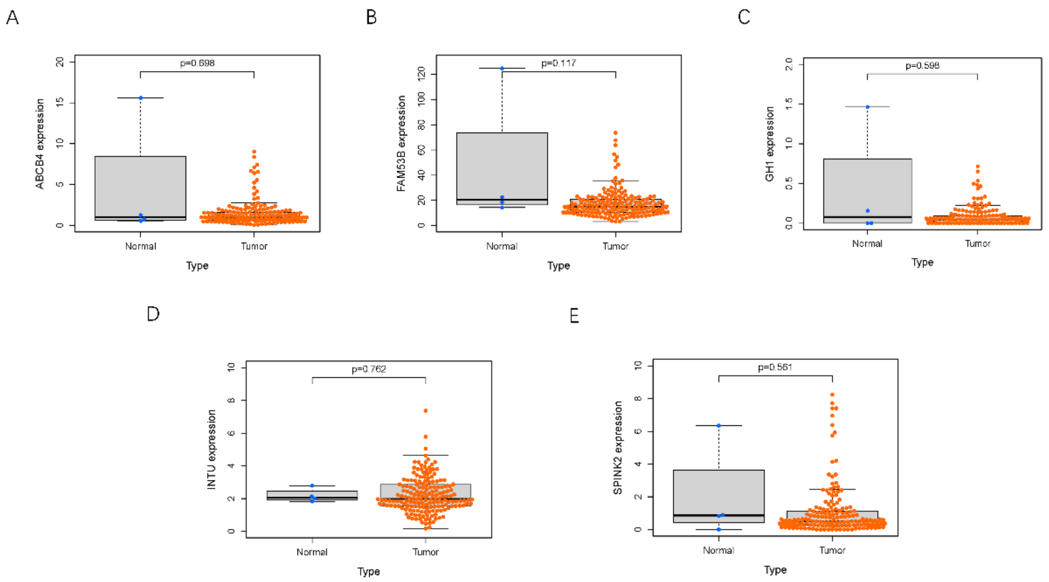

Figure S3: The mRNA expression level of hub genes in TCGA cohort. (A) ABCB4, (B) FAM53B, (C) GH1, (D) INTU, (E) SPINK2.

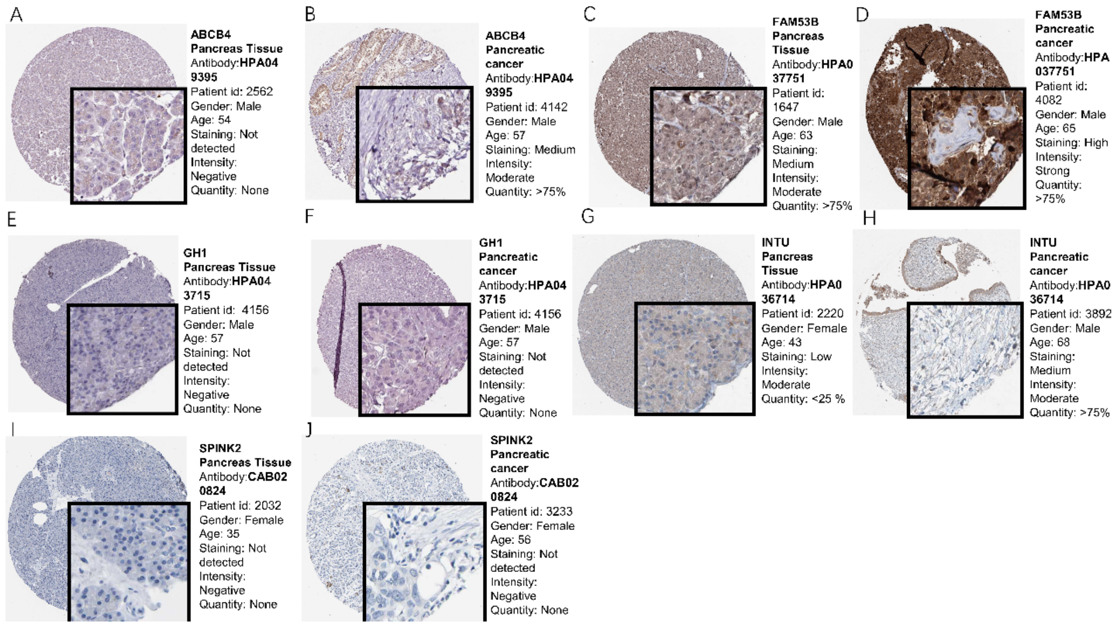

Figure S4: Differentially expressed proteins of ABCB4 in normal (A) and pancreatic cancer tissues (B) in the Human Protein Atlas database. Differentially expressed proteins of FAM53B in normal (C) and pancreatic cancer tissues (D) in the Human Protein Atlas database. Differentially expressed proteins of GH1 in normal (E) and pancreatic cancer tissues (F) in the Human Protein Atlas database. Differentially expressed proteins of INTU in normal (G) and pancreatic cancer tissues (H) in the Human Protein Atlas database. Differentially expressed proteins of SPINK2 in normal (I) and pancreatic cancer tissues (J) in the Human Protein Atlas database.

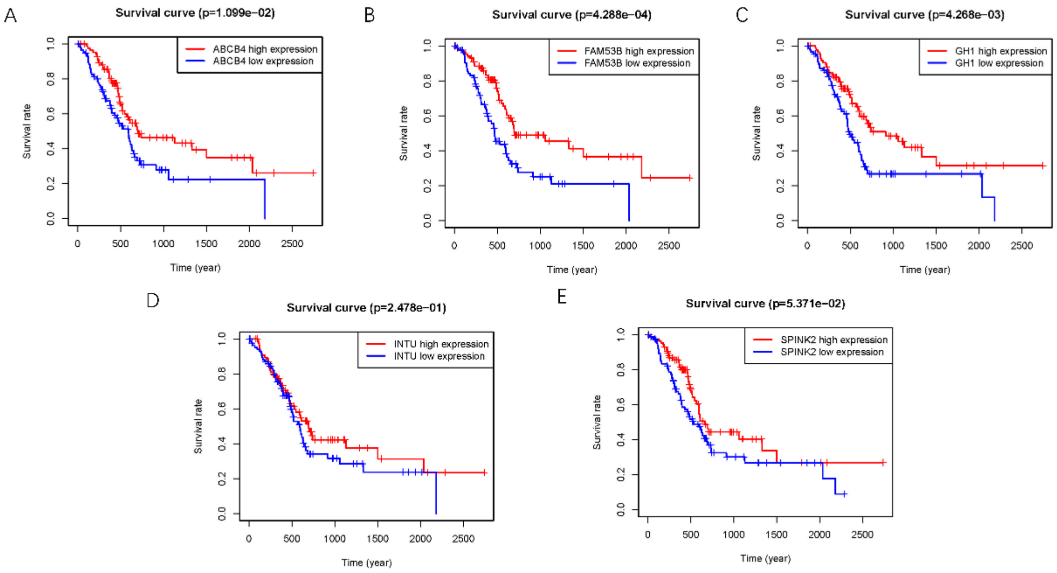

Figure S5: Survival analysis between high- and low- expression groups of hub genes. (A) ABCB4, (B) FAM53B, (C) GH1, (D) INTU, (E) SPINK2.

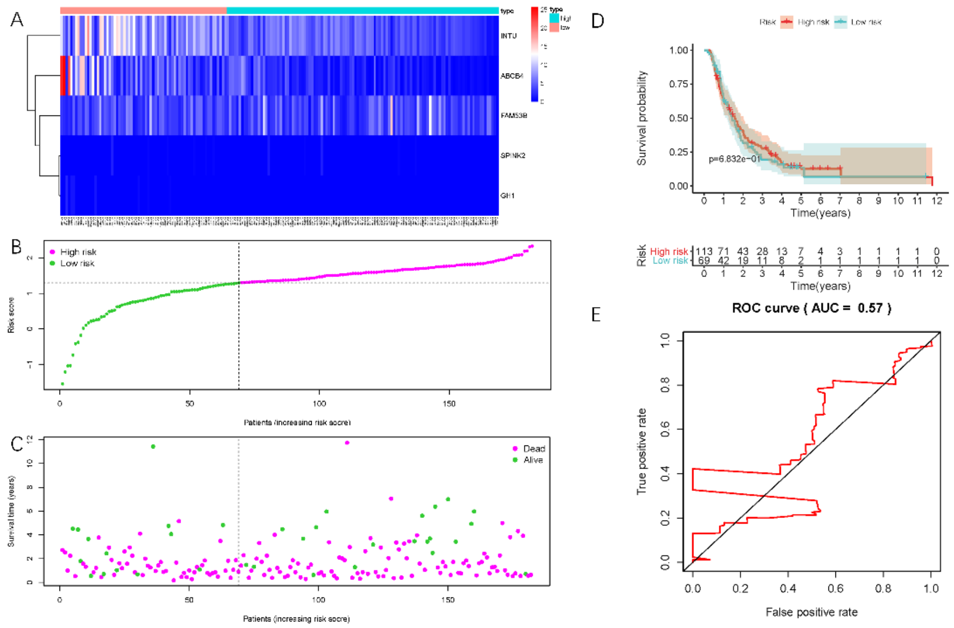

Figure S6: Confirmation of prognostic risk scores in the ICGC cohort. (A) Heatmap of the 5 hub genes expression in PDAC. The color from red to green shows a trend from high expression to low expression. (B) Distribution of model risk score. (C) The survival status and duration of PDAC patients. (D) Kaplan–Meier curve analysis presenting difference of overall survival between the high-risk and low-risk subgroups. (E) ROC analysis of the risk scores for prognosis prediction.

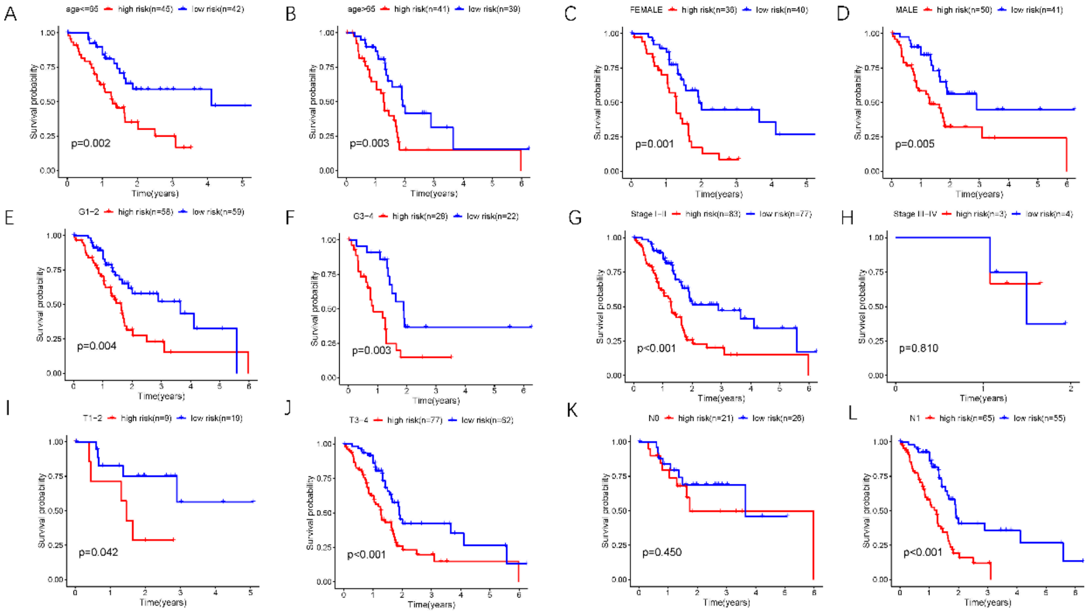

Figure S7: Kaplan–Meier survival analysis for multiple HCC subgroups according to the risk signature stratified by clinical variables. (A-B) Age. (C-D) Gender. (E-F) Tumor grade. (G-H) Stage. (I-J) T status. (K) N status. (L) M status.

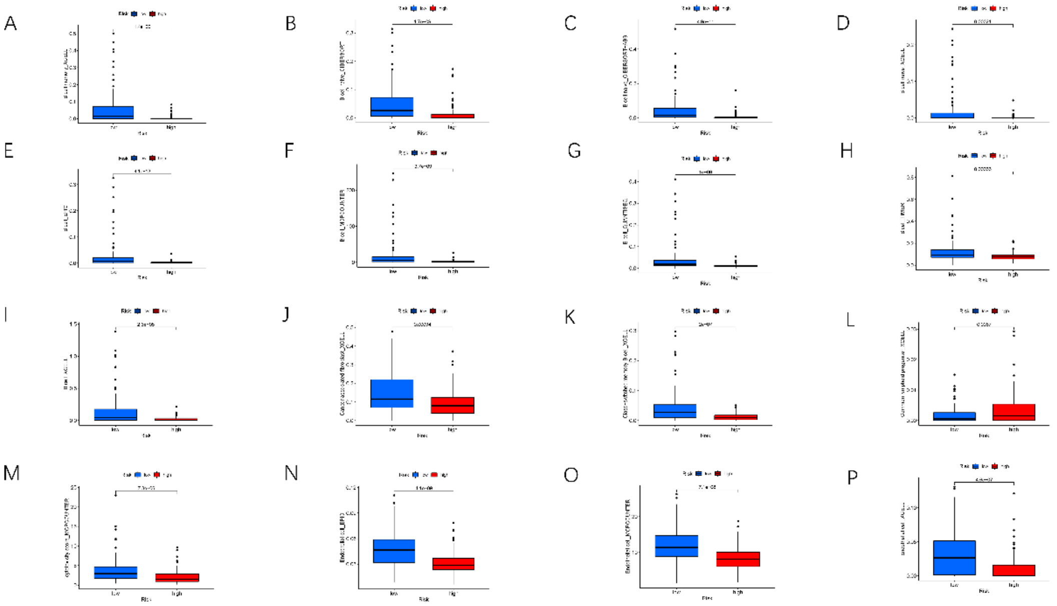

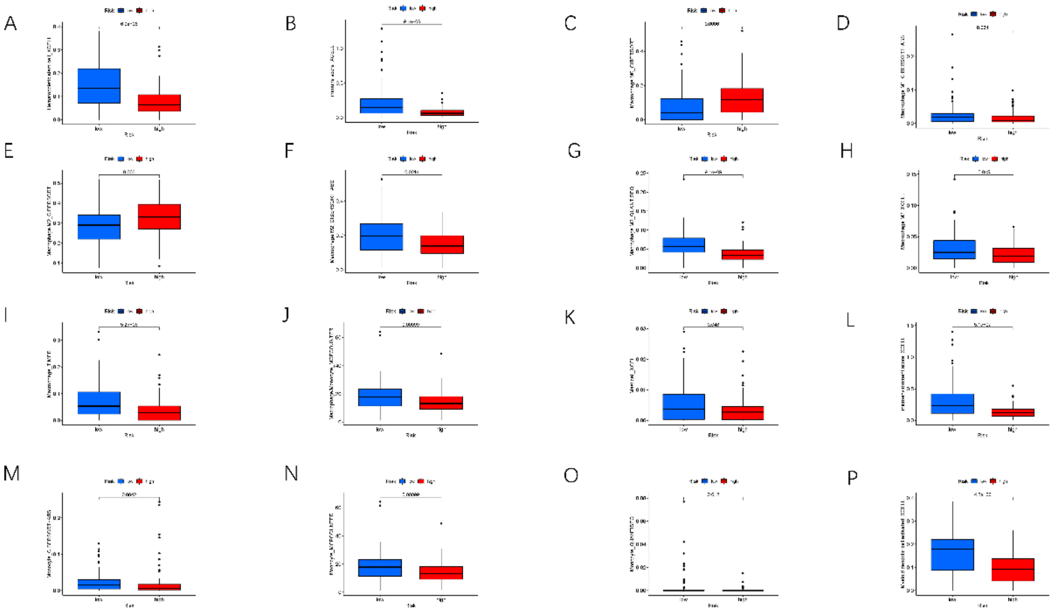
A
B
C
D
E
F
G
H
I
J
K
L
M
N
O
P

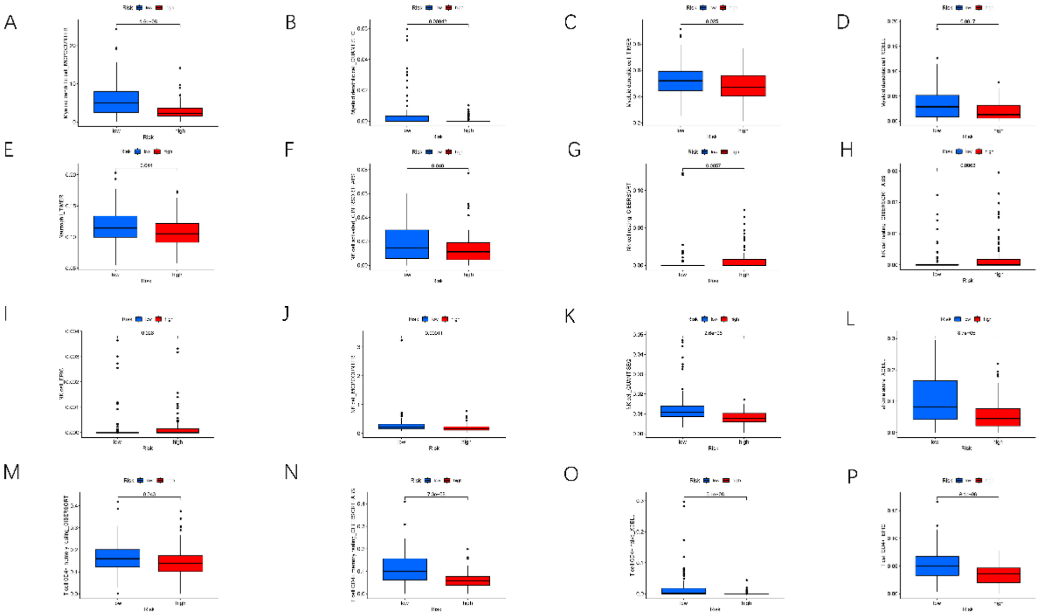
A
B
C
D
E
F
G
H
I
J
K
L
M
N
O
P

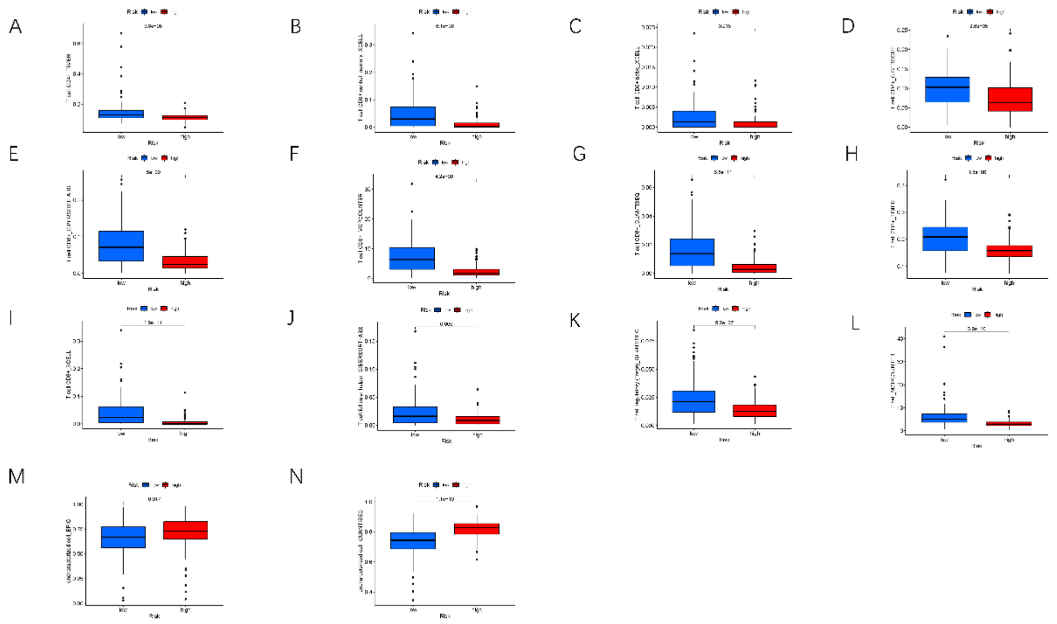

Figure S8-S11: The representative results of the evaluation of tumor infiltrating immune cells with risk signature.

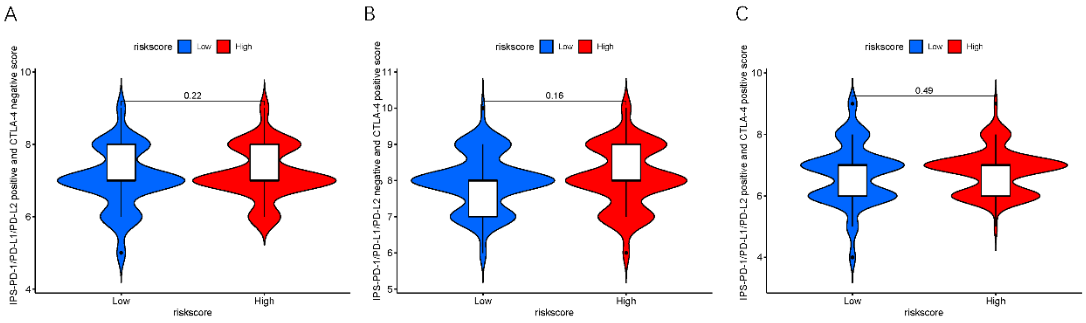

Figure S12. Prediction of Immunotherapeutic Response.

(A) IPS–CTLA4 blocker score distribution plot. (B) IPS–PD1/PDL1/PDL2 blocker score distribution plot. (C) IPS–CTLA4 and PD1/PDL1/PDL2 blocker score distribution plot.

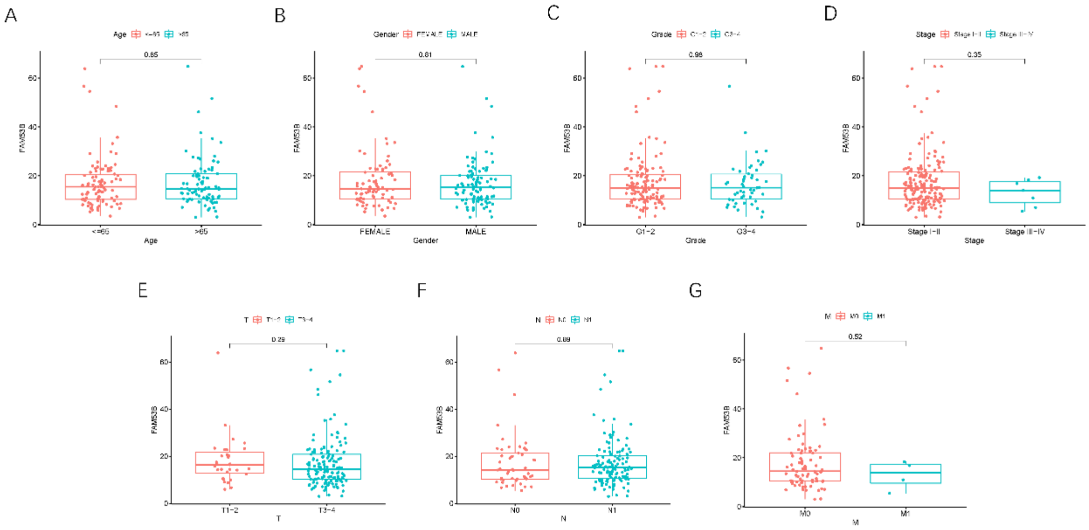

Figure S13. The clinical significance of FAM53B in PDAC. Distribution of risk score in distinct clinical variables subtypes. (A) Age, (B) Gender, (C) WHO grade, (D) clinical stage, (E) T status, (F) N status and (G) M status.
